# Integration of Wet-Lab Measures, Milk Infrared Spectra, and Genomics to Improve Difficult-to-Measure Traits in Dairy Cattle Populations

**DOI:** 10.3389/fgene.2020.563393

**Published:** 2020-09-29

**Authors:** Alessio Cecchinato, Hugo Toledo-Alvarado, Sara Pegolo, Attilio Rossoni, Enrico Santus, Christian Maltecca, Giovanni Bittante, Francesco Tiezzi

**Affiliations:** ^1^Department of Agronomy, Food, Natural Resources, Animals and Environment, University of Padova, Padua, Italy; ^2^Department of Genetics and Biostatistics, National Autonomous University of Mexico, Mexico City, Mexico; ^3^Italian Brown Breeders Association, Bussolengo, Italy; ^4^Department of Animal Science, North Carolina State University, Raleigh, NC, United States

**Keywords:** high-throughput phenotyping, Fourier-transformed infrared spectroscopy, genetic parameters, genomic predictions, dairy cattle, single-step GBLUP

## Abstract

The objective of this study was to evaluate the contribution of Fourier-transformed infrared spectroscopy (FTIR) data for dairy cattle breeding through two different approaches: (i) estimating the genetic parameters for 30 measured milk traits and their FTIR predictions and investigating the additive genetic correlation between them and (ii) evaluating the effectiveness of FTIR-derived phenotyping to replicate a candidate bull’s progeny testing or breeding value prediction at birth. Records were available from 1,123 cows phenotyped using gold standard laboratory methodologies (LAB data). This included phenotypes related to fine milk composition and milk technological characteristics, milk acidity, and milk protein fractions. The dataset used to generate FTIR predictions comprised 729,202 test-day records from 51,059 Brown Swiss cows (FIELD data). A first approach consisted of estimating genetic parameters for phenotypes available from LAB and FIELD datasets. To do so, a set of bivariate animal models were run, and genetic correlations between LAB and FIELD phenotypes were estimated using FIELD information obtained at the population level. Heritability estimates were generally higher for FIELD predictions than for the corresponding LAB measures. The additive genetic correlations (r_*a*_) between LAB and FIELD phenotypes had different magnitudes across traits but were generally strong. Overall, these results demonstrated the potential of using FIELD information as indicator traits for the indirect genetic improvement of LAB measures. In the second approach, we included genotype information for 1,011 cows from the LAB dataset, 1,493 cows from the FIELD dataset, 181 sires with daughters in both LAB and FIELD datasets, and 540 sires with daughters in the FIELD dataset only. Predictions were obtained using the single-step GBLUP method. A four fold cross-validation was used to assess the predictive ability of the different models, assessed as the ability to predict masked LAB records from daughters of progeny testing bulls. The correlation between observed and predicted LAB measures in validation was averaged over the four training-validation sets. Different sets of phenotypic information were used sequentially in cross-validation schemes: (i) LAB cows from the training set; (ii) FIELD cows from the training set; and (iii) FIELD cows from the validation set. Models that included FIELD records showed an improvement for the majority of traits. This study suggests that breeding programs for difficult-to-measure traits could be implemented using FTIR information. While these programs should use progeny testing, acceptable values of accuracy can be achieved also for bulls without phenotyped progeny. Robust calibration equations are, deemed as essential.

## Introduction

In the omics- era, an emerging field of research is represented by phenomics, which is the study of phenotypes on a genome-wide scale (Bilder et al., 2009; Houle et al., 2010). In animal breeding, the advance in high-throughput genomics has increased the need for simple, fast, accurate, and high-throughput phenotyping technologies. Fourier-transformed infrared spectroscopy (FTIR), including part of near- and mid-infrared (NIR and MIR) electromagnetic radiations, is a versatile and cost-effective analytical tool to collect individual data for monitoring traditional and novel milk traits in dairy cattle (Boichard and Brochard, 2012). For many years, milk composition traits such as fat and protein content, as well as lactose, urea, and casein content, have been routinely estimated by FTIR spectroscopy (Barbano and Clark, 1989). More recently, infrared technology has also been proposed as an alternative method for the quantification of difficult- or expensive-to-measure milk phenotypes including protein fractions, fatty acids, and minerals as well as milk coagulation properties (MCP), cheese yield, and curd nutrient recoveries (Soyeurt et al., 2006a, b, 2011; Ferragina et al., 2013; Cecchinato et al., 2015; Sanchez et al., 2018). In addition, FTIR data has been shown to be a potentially valuable tool for predicting health and reproductive phenotypes (Belay et al., 2017; Toledo-Alvarado et al., 2018), as well as residual feed intake, dry matter intake (DMI), and methane emissions (Bittante and Cipolat-Gotet, 2018; Dórea et al., 2018).

Within the animal breeding context, studies have shown the potential for using FTIR predictions as indicator traits of novel phenotypes like MPC, fatty acid profiles, and other milk components (Cecchinato et al., 2009; Rutten et al., 2011; Bonfatti et al., 2017). Multi-trait prediction allows simultaneous use of information from relatives and from different traits (Henderson and Quaas, 1976). It has been demonstrated that, using different databases, breeds, and traits, the effectiveness of FTIR calibrations to provide novel phenotypes exploitable in indirect selective breeding relies on the magnitude of heritability of FTIR predictions, and the additive genetic correlation between predictions and the measured traits (i.e., gold standard/breeding targets). Although the predictive ability of FTIR data is moderate for some traits, the genetic response achievable using FTIR predictions as indicator traits may be equal to or slightly lower than the response achievable from direct measurements of traits are utilized (Cecchinato et al., 2009; Rutten et al., 2010).

Besides the infrared technology, genome-wide prediction using the single-step approach has also been recognized as an important tool to predict phenotypes (Lee et al., 2008; Aguilar et al., 2010). The key principle for all these applications is the simultaneous estimation of all genome-wide marker effects based on a reference population with known phenotypes. Within this framework, the accuracy of prediction might also benefit from the added value of including genomic information in multi-trait prediction models, which have been shown to have better performances compared to single-trait models (Calus and Veerkamp, 2011; Guo et al., 2014; Karaman et al., 2018). While dairy cattle genetic improvement has historically hinged on progeny testing, genomic selection has made available high-accuracy breeding value predictions for candidate bulls at birth. This could mean that the traditional progeny testing is no longer required, provided that these early breeding value predictions are reliable. In this context, we hypothesized that multi-trait genomic prediction models applied to indicator traits estimated from routinely collected FTIR data, and their corresponding gold-standard measured traits could represent a viable option to evaluate the contribution of FTIR data collection for dairy cattle breeding. Cross-validation could be used to test the predictive ability of models that include or do not include FTIR-predicted phenotypes, to replicate a candidate bulls’ progeny testing or prediction of breeding value at birth.

Therefore, the overall objective of this study was to test the value of FTIR predictions from field data (FIELD) for the genetic improvement of difficult-to-measure traits in dairy cattle. Steps to address this objective were (i) to infer (co)variance components, heritabilities, and additive genetic correlations between 30 LAB-measured and FIELD-predicted phenotypes, divergent in terms of biological meaning, variability, and heritability, related to fine milk composition and milk technological characteristics [traditional MCP, curd firming (CF) traits, cheese yields and recoveries of nutrients, milk acidity and milk protein fractions] and (ii) to use bivariate single-step GBLUP for evaluating the predictive ability of FTIR-derived phenotyping for these traits by using different phenotyping and genotyping strategies.

## Materials and Methods

### Ethics Statement

This study did not require any specific ethics permit. The cows sampled belonged to commercial private herds and were not experimentally manipulated. Milk samples were collected during routine milk recording coordinated by technicians from the Breeders Federations of Trento Province (FPA, Trento, Italy) and of Alto Adige/Südtirol (Associazione Provinciale delle Organizzazioni Zootecniche Altotesine/Vereinigung der Südtiroler Tierzuchtverbände, Bolzano/Bozen, Italy).

### Data Structure

In this study, we used two sets of data collected on Brown Swiss cows: (i) a LAB dataset in which laboratory measurements and spectra data for phenotypes related to milk quality and cheese-making were available to develop calibration equations and (ii) a FIELD-FTIR dataset for testing field prediction at the population level. A subset of the FIELD-FTIR dataset including only first lactation records was used for estimating (co)variance components (FIELD_*lact*1_); the whole database was instead used for the genomic analyses (FIELD). The data structure is summarized in Table 1.

**TABLE 1:** Summary of data structure for laboratory (LAB) measures, after editing, and Fourier transform infrared predictions (FIELD).

Item	LAB	FIELD_*lact*1_^1^	FIELD^2^
Animals	1,123	39,833	51,059
Records	1,123	235,372	729,202
Herds	83	2,494	2,607
Animals in the pedigree	6,526	97,933	136,332
Number of generations	5	5	13
Sires	266	1,210	1,835
Dams	1,044	29,716	38,449

*^1^FIELD_lact1_ = dataset limited to cows belonging to first lactation. ^2^FIELD = whole dataset.*

### LAB Dataset

The LAB data were part of the Cowability/Cowplus projects. Individual milk samples from 1,200 Brown Swiss cows from 85 herds located in the Alpine province of Trento (Italy) were collected. Details of the animals used in this study and characteristics of the area are reported in Mele et al. (2016). Data on the cows, herds, and single test-day milk yield were provided by the Superbrown Consortium of Bolzano and Trento (Italy), and pedigree information was supplied by the Italian Brown Swiss Cattle Breeders Association (Verona, Italy).

### FTIR Spectra Data

Individual milk samples were analyzed using a MilkoScan FT6000 (Foss Electric, Hillerød, Denmark). The spectrum covers from the short-wavelength infrared (SWIR, also known as NIR or IR-B), through the medium-wavelength infrared (MWIR, also known as MIR), to the long-wavelength infrared (LWIR, or LIR) regions with 1,060 spectral points from wavenumber 5,010 to 925 cm^–1^, which correspond to wavelengths ranging from 1.99 to 10.81 μm and frequencies ranging from 150.19 to 27.73 THz. Spectra were expressed as absorbance calculated as log(1/transmittance). Two spectral acquisitions were carried out for each sample (collected during the evening milking), and the results were averaged before data analysis (Ferragina et al., 2015).

### Phenotypes

#### Traditional Milk Coagulation Properties

Measures of MCP were obtained using two different instruments: a Formagraph (Foss Electric A/S) and an Optigraph (Ysebaert SA, Frépillon, France) according to Cecchinato et al. (2013). Briefly, milk samples (10 mL) were heated to 35°C and 200 μL of a rennet solution (Hansen Standard 160, with 80 ± 5% chymosin and 20 ± 5% pepsin; 160 international milk clotting units/mL; Pacovis Amrein AG, Bern, Switzerland), diluted to 1.6% (wt/vol) in distilled water, was added at the beginning of the analysis. The time of analysis was extended up to 90 min after rennet addition. Rennet coagulation time (RCT) was defined as the time (min) from the addition of enzyme to the beginning of coagulation, k_20_ (min) was defined as the interval from RCT to the time at which a curd firmness of 20 mm was obtained, and a_30_ and a_45_ (mm) were measurements of curd firmness at 30 and 45 min after rennet addition, respectively.

#### Modeling the Curd Firmness

A set of parameters of CF at time *t* (CF_*t*_) was estimated, and details are described in Bittante et al. (2015). Estimated parameters included rennet coagulation time (RCT_*eq*_, min), estimated from the CF_*t*_ equation; potential asymptotical curd firmness (CF_*P*_, mm), representing the maximum potential curd firmness after infinite time in the absence of syneresis; curd-firming rate constant (k_*CF*_, %/min), which is a measure of the rate of CF; syneresis rate constant (k_*SR*_, %/min); maximum curd firmness (CF_*max*_, mm); and time to CF_*max*_ (t_*max*_, min).

#### Milk Acidity

The milk pH was measured using a Crison Basic 25 electrode (Crison, Barcelona, Spain).

#### Cheese Yields and Curd Nutrient Recoveries

To assess cheese-making properties, milk samples were processed according to all the steps of the cheese-making practice used in artisanal commercial dairies for producing a traditional whole milk cheese described by Bittante et al. (2014). Briefly, 1,500 mL of milk was heated to 35°C in a stainless steel microvat, supplemented with thermophilic starter culture, and mixed with rennet. The resulting curd from each vat was double-cut and heated for 30 min to 55°C, drained, shaped in wheels, pressed, salted, and weighed. The whey was drained from the curd, weighted, and analyzed for fat, protein, lactose, and total solid content using FT2 (Foss Electric A/S, Hillerød, Denmark). Three cheese yield (CY) traits were calculated expressing the weight (wt) of fresh curd (%CY_*CURD*_), of curd DM (%CY_*SOLIDS*_), and of water retained in the curd (%CY_*WATER*_) as a percentage of the weight of milk processed. Four recovery (REC) traits were calculated as proportions of nutrients and energy of the milk retained in the curd (REC_*SOLIDS*_, REC_*FAT*_, REC_*PROTEIN*_, and REC_*ENERGY*_ calculated as the % ratio between the nutrient in curd and the corresponding nutrient in processed milk). The energy within the curd was calculated as the difference between energy in the milk and in the whey (NRC, 2001).

#### Milk Proteins

Concentrations of the major casein (CN) fractions (α_*S1–*_CN, α_*S2–*_CN, β-CN, and κ-CN) and whey proteins, β-lactoglobulin (β-LG), and alpha-lactalbumin (α-LA) were determined using a validated reversed phase high-performance liquid chromatography (RP-HPLC) method (Bonfatti et al., 2008). Each protein fraction was expressed as a percentage of the milk total nitrogen (N) content.

#### Data Editing

Only records with spectra and measured phenotypes available were kept. After data editing and removing observations outside three standard deviations, the final number of records and phenotypes used in subsequent analyses varied from 770 to 1,120 depending on the trait as reported in Table 2.

**TABLE 2:** Descriptive statistics for laboratory (LAB) measures and Fourier transform infrared predictions (FIELD) for the investigated traits.

Trait^1^	LAB			FIELD_*lact*1_^2^			FIELD^3^		
	*N*	Mean	*SD*	*N*	Mean	*SD*	*N*	Mean	*SD*
**Traditional MCP**									
RCT, min	1,096	19.8	5.38	232,660	20.47	4.76	712,420	19.98	4.81
k_20_, min	1,073	5.34	2.43	232,775	5.59	1.68	710,826	5.59	1.66
a_30_, mm	1,051	29.29	10.97	232,702	27.3	8.53	713,759	27.92	8.59
a_45_, mm	1,096	33.28	8.00	232,869	33.31	4.06	714,254	32.78	4.03
**Curd firming**									
RCT_eq_, min	1,093	20.62	5.41	233,828	21.23	5.17	718,385	20.86	5.18
CF_p_, mm	1,105	49.89	9.43	234,065	49.21	5.95	723,632	49.37	6.14
k_CF_, % × min^–1^	1,104	12.83	3.91	233,641	12.12	2.61	721,173	12.84	2.70
k_SR_, % × min^–1^	1,102	1.21	0.43	233,568	1.14	0.23	721,261	1.21	0.25
C_max_, mm	1,105	37.23	7.03	234,106	37.04	4.53	723,364	36.80	4.68
t_max_, min	1,071	40.34	9.85	233,779	42.76	8.74	720,164	41.17	8.88
**Optigraph**									
RCT, min	787	18.88	4.11	232,856	18.77	3.29	712,568	18.80	3.35
k_20_, min	770	8.04	2.62	232,628	8.09	2.15	709,025	8.41	2.09
a_30_, mm	782	26.6	10.6	232,561	25.91	8.34	713,482	25.54	8.31
a_45_, mm	786	41.03	11.04	232,783	40	8.67	713,992	39.65	8.63
**Acidity**									
pH	1,112	6.64	0.08	232,772	6.63	0.07	715,433	6.65	0.06
**Cheese yields, %**									
CY_CURD_	1,120	15.04	1.9	234,247	14.92	1.64	722,328	14.95	1.65
CY_SOLIDS_	1,101	7.19	0.9	233,860	7.11	0.84	719,915	7.13	0.86
CY_WATER_	1,112	7.80	1.29	234,317	7.9	1.03	724,431	7.77	1.04
**Recoveries, %**									
REC_PROTEIN_	1,112	78.09	2.44	233,940	78.81	2.18	720,358	78.25	2.28
REC_FAT_	1,083	89.97	3.27	233,818	89.39	2.92	716,200	89.48	2.95
REC_SOLIDS_	1,115	52.00	3.55	233,973	51.21	3.07	721,441	51.66	3.18
REC_ENERGY_	1,101	67.25	3.28	233,630	66.58	2.89	719,822	67.05	3.00
**N fractions, % total N**									
Caseins	1,097	77.97	1.2	233,395	78.25	1.41	700,206	77.88	1.36
β-CN	1,098	32.12	2.42	233,861	32.86	1.60	721,345	32.22	1.69
κ-CN	1,087	9.53	1.33	234,358	9.26	1.50	713,219	9.44	1.49
α_S1_-CN	1,096	25.71	1.69	235,058	25.80	0.71	724,579	25.72	0.75
α_S2_-CN	1,091	9.17	1.05	234,457	9.12	0.58	723,390	9.15	0.59
Whey proteins	1,094	11.06	1.61	234,276	11.08	1.16	722,788	10.99	1.18
β-LG	1,091	8.70	1.47	234,252	8.71	1.33	721,472	8.63	1.36
α-LA	1,097	2.37	0.49	232,535	2.38	0.46	713,999	2.34	0.46

*^1^RCT = rennet coagulation time; k_20_ = curd firming rate as the time to a curd firmness of 20 mm; a_30 (45)_ = curd firmness at 30 (45) min from rennet addition; RCT_eq_ = rennet coagulation time estimated using the equation; CF_P_ = asymptotic potential curd firmness; k_CF_ = curd firming instant rate constant; k_SR_ = syneresis instant rate constant; CF_max_ = maximum curd firmness achieved within 45 min; t_max_ = time at achievement of CF_max_; %CY_CURD_ = weight of fresh curd as percentage of weight of milk processed; %CY_SOLIDS_ = weight of curd solids as percentage of weight of milk processed; %CY_WATER_ = weight of water curd as percentage of weight of milk processed; REC_PROTEIN_ = protein of the curd as percentage of the protein of the milk processed; REC_FAT_ = fat of the curd as percentage of the fat of the milk processed; REC_SOLIDS_ = solids of the curd as percentage of the solids of the milk processed; REC_ENERGY_ = energy of the curd as percentage of energy of the milk processed; true protein nitrogen (N) and milk N fractions are expressed as percentage of total milk N; β-CN (β-casein), κ-CN (κ-casein), αs_1_-CN (αs_1_-casein), αs_2_- CN (αs_2_-casein); caseins: Σ(β-CN+κ-CN+ αs_1_-CN+αs_2_-CN); β-LG (β-lactoglobulin), α-LA (α-lactalbumin), whey proteins: Σ(β-LG+ α-LA). ^2^FIELD_lact1_ = dataset limited to cows belonging to first lactation. ^3^FIELD = whole dataset.*

### Field Data

#### Spectra Data

The FIELD data for our study were provided by the Breeders Federations of Trento Province (FPA, Trento, Italy) and of Alto Adige/Südtirol (Associazione Provinciale delle Organizzazioni Zootecniche Altotesine/Vereinigung der Südtiroler Tierzuchtverbände, Bolzano/Bozen, Italy) as part of the Cowability/Cowplus projects. All milk samples were analyzed using a MilkoScan FT6000 (Foss Electric, Hillerød, Denmark). Spectra characteristics, in terms of wavelengths and way of expression (absorbance), were the same as for the LAB data. Spectra were collected between 2010 and 2017.

#### Spectra Editing

A preliminary analysis was carried out to identify the outlier spectra based on the Mahalanobis distance from the first five principal component scores. The probability level for the chi-squared distribution of a sample’s Mahalanobis distance was calculated from the incomplete gamma function with five degrees of freedom (Toledo-Alvarado et al., 2018). Samples with P < 0.01 were removed from the dataset. To overcome spectral variations, the absorbance values for every wavelength were centered to a null mean and standardized to a unit sample variance within year periods. Records without parity number, date of calving, animal ID, or pedigree information were also removed. If a cow had predictions from both LAB and FIELD datasets, the FIELD prediction records of that specific cow were deleted. This latter editing step was performed for the following reasons: (i) to avoid an overestimation of additive genetic correlations; (ii) to force the connection between LAB and FIELD data only through the additive genetic effect; and (iii) to avoid EBV inflation for cows with both LAB and FIELD records.

To detect outliers among the predicted phenotypes, a mixed model was fitted for each trait including the fixed non-genetic effects of (i) the stage of lactation (12 monthly classes), (ii) the combined effect of herd (∼2,460 levels), year of the test day (2010–2017), and two seasons of calving (April to September and October to March) (∼22,200 levels). The permanent environmental effects (∼39,600) and animal additive genetic effects were included as random terms.

The residuals of phenotypes outside 3 standard deviations were considered as outliers.

### Genotypes

The pool of genotyped individuals consisted of (i) 1,011 LAB cows genotyped with the Illumina BovineSNP50 v.2 BeadChip (Illumina, Inc., San Diego, CA, United States; 54,000 SNPs); (ii) 1,463 FIELD cows which were genotyped with the BovineLD v2.0 BeadChip (Illumina, Inc., San Diego, CA, United States; 7,931 SNPs); (iii) 181 sires with both LAB and FIELD daughters genotyped with the Illumina BovineSNP50 v.2 BeadChip or the Illumina Bovine HD BeadChip (Illumina, Inc., San Diego, CA, United States; 777,000 SNPs); and (iv) 540 sires with FIELD daughters only genotyped as (v). The software FImpute (Sargolzaei et al., 2014) was used for imputation and all individuals were imputed to the BovineSNP50 v.2 BeadChip panel. A total number of 3,195 genotyped individuals were available for this study.

Marker editing was performed using the preGSf90 software (Misztal et al., 2002). Markers were excluded where the call rate was below 95%, the minor allele frequency was below 5%, and/or there was significant deviation from the Hardy–Weinberg equilibrium (P < 10^–6^). SNPs mapped to the sex chromosomes or with unknown position on the genome were also removed. After editing, the number of markers available for analyses was 37,093.

### Statistical Analyses

#### Calibration Equations

Separate models were fitted for each trait considered. We used a Bayesian model (BayesB model) implemented in BGLR (de los Campos and Perez-Rodriguez, 2014), as previously described by Ferragina et al. (2015). Phenotypes in LAB (i.e., the calibration dataset) were regressed on standardized spectra covariates using the linear model:

$$y_{i}=\beta_{0}+\sum_{j=1}^{1,060}x_{ij}\beta_{j}+\varepsilon_{i,}$$

where β_0_ is an intercept, {*x*_*ij*_} are standardized FTIR spectra-derived wavelength data(*j* = 1,⋯1,060), _β_*j*__ are the effects of each of the wavelengths, and ε_*i*_ are model residuals assumed to be independent and identically distributed, with normal distribution centered at zero and variance $\sigma_{\varepsilon }^{2}$. Models were applied with 100,000 iterations and 20,000 chains discarded as burn in. The editing and analysis were conducted using R software (R Core Team, 2018). To evaluate predictive performance, the coefficient of correlation for the calibration model developed on LAB measures and used for FIELD predictions was calculated for each trait (r_*C_LAB*_). The calibration equations obtained with this procedure in LAB data were then applied to the spectral population data (FIELD) in order to obtain FIELD predictions for all traits of concerns.

#### Genetic Analyses: (Co)Variance Components Estimation Between Measured (LAB) and Predicted (FIELD) Phenotypes

The (co)variance components were estimated using REMLF90 and AIREMLF90 (Misztal et al., 2002), considering LAB measures and FIELD predictions as distinct traits. The connection between the two datasets was guaranteed by 266 sires, 1,044 dams, 94 sires of sires, and 372 sires of dams in common between LAB and FIELD datasets (Table 1). To save time and improve convergence of models, for this first approach, the FIELD dataset has been limited to cows belonging to first lactation (FIELD_*lact*1_).

The program was run until a convergence criterion of 1^*e*−10^ was reached.

The model for LAB-measures was

$$y=Xb+Z_{a}a+e,\left(1\right)$$

where **y** is the vector of observations for traits of concern; **b, a,** and **e** correspond to the vector of fixed non-genetic effects, random animal additive genetic effects, and random residual effects, respectively; **X** and **Z_*a*_** are the incidence matrices relating each observation in **y** to **b** and **a**. The non-genetic fixed effects included in the model were (i) the DIM of each cow within parity (60 levels) and (ii) herd-test day (83 levels). The random terms were animal additive genetic effects and residual effects. The pedigree file included all phenotyped animals and their ancestors (∼6,500 animals).

$$\mathrm{Heritability}\mathrm{was}\mathrm{computed}\mathrm{as}h^{2}=\frac{\sigma_{a}^{2}}{\sigma_{a}^{2}+\sigma_{e}^{2}}$$

where $\sigma_{a}^{2}$ and $\sigma_{e}^{2}$ are the additive genetic and residual variances, respectively.

The model for FIELD_*lact*1_ records was

$$y=Xb+Z_{a}a+Z_{pe}pe+e,\left(2\right)$$

where **y** is the vector of observations for the traits of concern; b, a, pe, and e correspond to the vector of fixed non-genetic effects, random animal additive genetic effects, random permanent environmental effects, and random residuals effects, respectively; **X**, **Z_*a*_**, and **Z_*pe*_** are the incidence matrices relating each observation in y, a, b, and pe, respectively. The fixed non-genetic effects were (i) the stage of lactation (12 monthly classes) and (ii) the combined effect of herd (∼2,460 levels), year of the test-day (2010–2017), and season of calving (April to September and October to March) (∼22,200 levels). The random terms were permanent environmental effects (∼39,600), animal additive genetic effects, and residual effects. The pedigree file included all phenotyped animals and their ancestors (∼97,900 animals). Only first lactation records were utilized to avoid convergence problems. The residuals were considered uncorrelated across the two traits (LAB and FIELD_*lact*1_ predictions).

$$\mathrm{Heritability}\mathrm{was}\mathrm{computed}\mathrm{as}{}_{h^{2}=\frac{\sigma_{a}^{2}}{\sigma_{a}^{2}+\sigma_{\mathrm{pe}}^{2}+\sigma_{e}^{2}}}$$

where $\sigma_{a}^{2}$, $\sigma_{pe}^{2}$, and $\sigma_{e}^{2}$ are the additive genetic, permanent environmental, and residual variances, respectively.

For each trait, the additive genetic correlation between LAB and FIELD_*lact*1_ was estimated from the variance–covariance matrix of the random additive genetic effect as $r_{a}=\frac{\sigma_{a1,a2}}{\sigma_{a1}\times \sigma_{a2}}$ where σ_*a*1,*a*2_ is the additive genetic covariance between two traits, and σ_*a*1_ and σ_*a*2_ are the additive genetic standard deviations for traits 1 and 2, respectively.

#### Predictive Ability Estimated Using Genomic and Infrared Information

In order to assess the ability of FIELD data to predict LAB records as phenotypes of interest, a fourfold cross-validation was used. In this setting, the LAB phenotype is considered as the breeding goal while the FIELD record is its correlated trait. The models employed for both LAB and FIELD data were the same as for the variance component estimation, except for the stage of lactation effect in the FIELD dataset that was replaced with a stage of lactation by parity effect (5 parity classes, 72 levels in total), since the whole FIELD dataset was used (not restricting the records to first lactation).

Sires with both LAB and FIELD daughters were randomly assigned to four groups, evenly sized based on the number of LAB cows for each sire. Details on the number of records and cows in each set are reported in Supplementary Table S1. Cross-validation was performed using alternatively 3 groups for training (denoted with the suffix “t”) and one group for validation (denoted with the suffix “v”), for the scenarios that only included the training sets.

The cross-validation considered different training sets, either using LAB or FIELD data alone or combined: (i) model “LAB.t” included phenotypes available are from LAB cows that were daughters of the sires in the training set; (ii) model “FIELD.t” included phenotypes available from FIELD cows that were daughters of the sires in the training set; (iii) model “LAB.t + FIELD.t” included phenotypes from LAB and FIELD cows that were daughters of the sires in the training set; (iv) model “FIELD.t + FIELD.v” included phenotypes available are from all FIELD cows (no alternate masking of phenotypes was performed here); and (v) model “LAB.t + FIELD.t + FIELD.v” included phenotypes available from LAB cows that were daughters of the sires from the training set and all FIELD cows. Model “LAB.t” assesses the predictive ability when FIELD records are not collected, i.e., no use of FTIR data. Model “FIELD.t” evaluates the impact of FIELD phenotyping the daughters of the bulls in the reference population, with no LAB phenotypes included in the genetic evaluation. Model “LAB.t + FIELD.t” evaluates the impact of including FIELD phenotypes for the daughters of the bulls in the progeny test. In model “FIELD.t + FIELD.v,” progeny testing bulls have daughters phenotyped with FTIR data while in model “LAB.t + FIELD.t + FIELD.v,” the progeny testing bulls have daughters phenotyped with LAB data as well.

Accuracy of prediction was quantified as

$$acc_{x}=cor\left(y_{val,x,}^{LAB}\left(G\right)EBV_{val,x}^{LAB}\right)$$

where *acc*_*x*_ is the accuracy in the validation set *x* (*x* = 1,2,3,4), $y_{val,x}^{LAB}$ are the masked LAB records from cows in validation set *x*, $\left(G\right)EBV_{val,x}^{LAB}$ is the estimated genomic breeding value for LAB cows in validation set *x*, and *cor* refers to the Pearson correlation. Estimated genomic breeding values were calculated using the program BLUPf90 (Misztal et al., 2002) that combines pedigree-derived and SNP-derived genomic relationship matrices (Legarra et al., 2009). All 3,195 genotypes available for this study were included in the analysis.

For this part of the study, we did not use the variance components estimated in the previous step, but we tested different genetic correlation (covariance) values in order to find those that better maximized prediction accuracy. Firstly, estimates of variance components were obtained from single-trait models (Supplementary Table S2). Single-trait models were used in order to remove the inflation in the estimates due to genetic covariances. Then, different values of genetic correlation between LAB and FIELD phenotypes ranging from 0.1 to 1, with 0.1 step increases, were generated. Each prediction run was then repeated for the values of covariance generated by each value of assumed genetic correlation. This allowed the exploration of the predictive ability over all the potential values of genetic correlation.

## Results and Discussion

### Descriptive Statistics

Descriptive statistics for all investigated LAB-measured and FIELD-predicted traits are summarized in Table 2. Extensive description and discussion of the results for the LAB dataset have been reported in previous works, including the differences between MCP measured with FRM and OPT (Cecchinato et al., 2013; Bittante et al., 2015; Pegolo et al., 2018). Generally, we observed consistency between the mean values from LAB, FIELD_*lact*1_, and FIELD datasets for all the investigated traits. We only observed a slight loss of variability for all the FTIR-predicted phenotypes as demonstrated by the lower standard deviations, compared to for the observed traits.

### Variance Components and Heritability of Measured and Predicted Traits

#### Coagulation Traits and Milk Acidity

**FIGURE 1:**
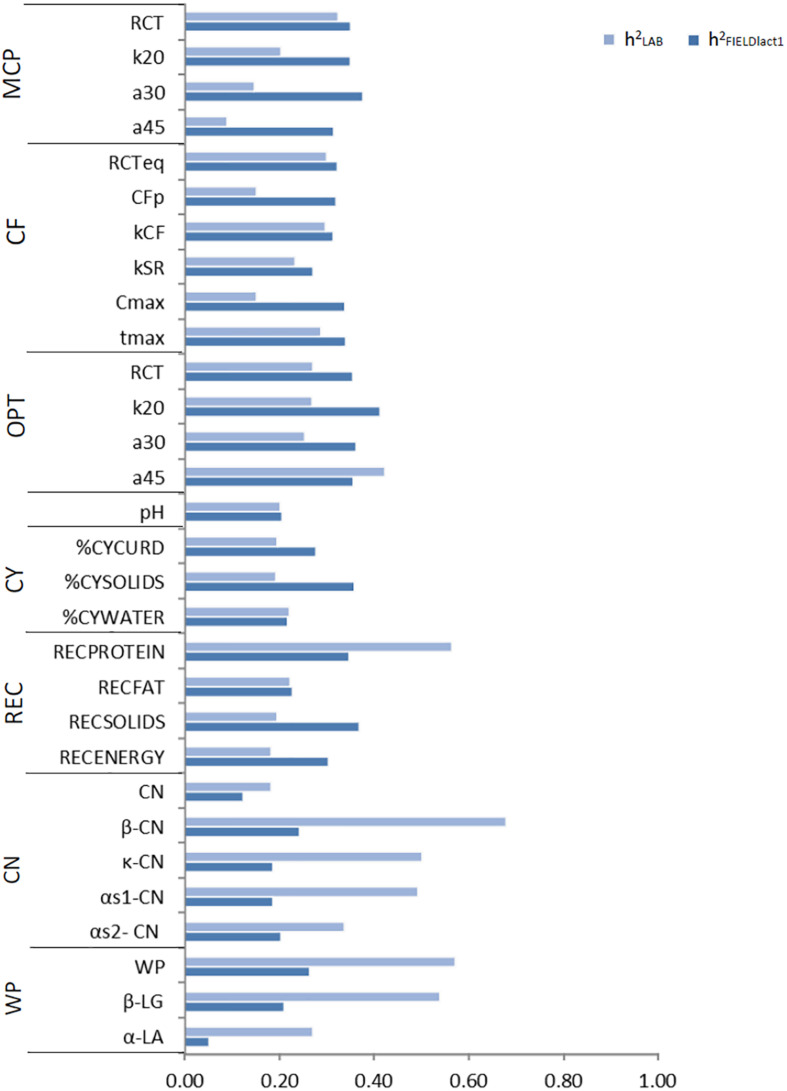
Estimates of heritability for LAB measures and FTIR predictions of the investigated traits using LAB and FIELD_*lact*1_ data. MCP = milk coagulation properties; RCT = rennet coagulation time; k_20_ = curd firming (CF) rate as the time to a curd firmness of 20 mm; a_30 (45)_ = curd firmness at 30 (45) min from rennet addition; RCT_*eq*_ = rennet coagulation time estimated using the equation; CF_*P*_ = asymptotic potential curd firmness; k_*CF*_ = curd firming instant rate constant; k_*SR*_ = syneresis instant rate constant; CF_*max*_ = maximum curd firmness achieved within 45 min; t_*max*_ = time at achievement of CF_*max*_; OPT = Optigraph; CY = cheese yield; %CY_*CURD*_ = weight of fresh curd as percentage of weight of milk processed; %CY_*SOLIDS*_ = weight of curd solids as percentage of weight of milk processed; %CY_*WATER*_ = weight of water curd as percentage of weight of milk processed; REC = recoveries; REC_*PROTEIN*_ = protein of the curd as percentage of the protein of the milk processed; REC_*FAT*_ = fat of the curd as percentage of the fat of the milk processed; REC_*SOLIDS*_ = solids of the curd as percentage of the solids of the milk processed; REC_*ENERGY*_ = energy of the curd as percentage of energy of the milk processed. ^1^True protein nitrogen (N) and milk N fractions are expressed as percentage of total milk N; β-CN (β-casein), κ-CN (κ-casein), αs_1_-CN (αs_1_-casein), αs_2_-CN (αs_2_-casein); caseins (CN): Σ(β-CN+κ-CN+ αs_1_-CN+αs_2_-CN); β-LG (β-lactoglobulin), α-LA (α-lactalbumin), whey proteins (WP): Σ(β-LG+ α-LA).

**FIGURE 2:**
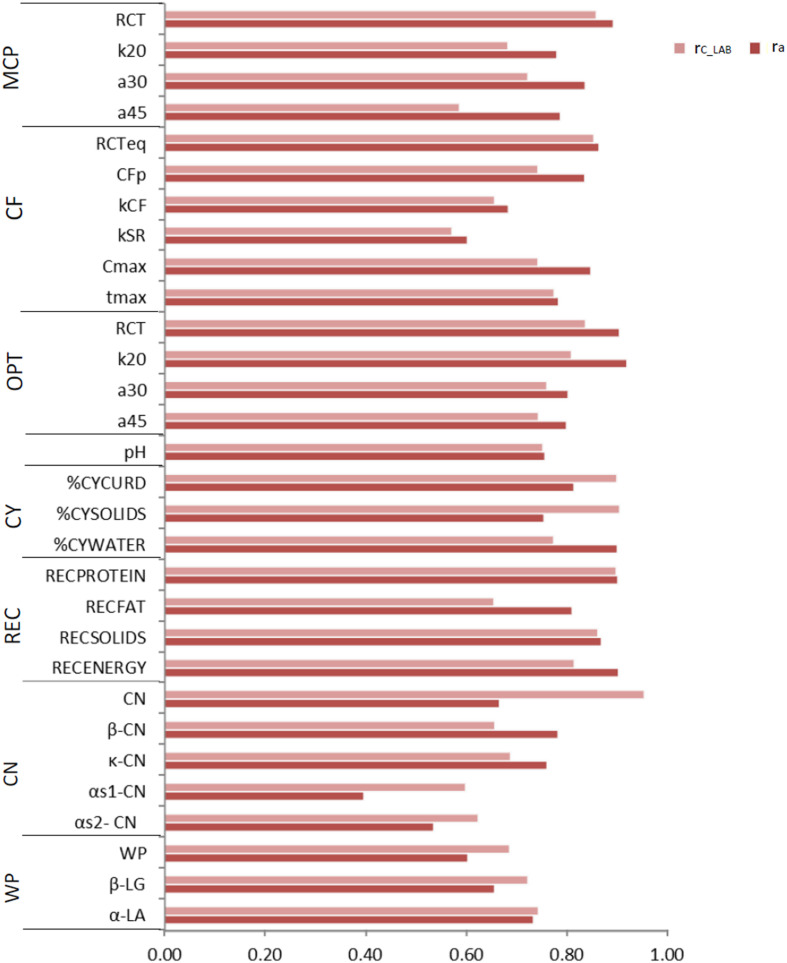
Coefficient of correlation of the calibration models used for infrared prediction (r_*C_LAB*_) and additive genetic correlations (r_*a*_) between LAB measures and FTIR predictions of the investigated traits using LAB and FIELD_*lact*1_ data. MCP = milk coagulation properties; RCT = rennet coagulation time; k_20_ = curd firming (CF) rate as the time to a curd firmness of 20 mm; a_30 (45)_ = curd firmness at 30 (45) min from rennet addition; RCT_*eq*_ = rennet coagulation time estimated using the equation; CF_*P*_ = asymptotic potential curd firmness; k_*CF*_ = curd firming instant rate constant; k_*SR*_ = syneresis instant rate constant; CF_*max*_ = maximum curd firmness achieved within 45 min; t_*max*_ = time at achievement of CF_*max*_; OPT = Optigraph; CY = cheese yield; %CY_*CURD*_ = weight of fresh curd as percentage of weight of milk processed; %CY_*SOLIDS*_ = weight of curd solids as percentage of weight of milk processed; %CY_*WATER*_ = weight of water curd as percentage of weight of milk processed; REC = recoveries; REC_*PROTEIN*_ = protein of the curd as percentage of the protein of the milk processed; REC_*FAT*_ = fat of the curd as percentage of the fat of the milk processed; REC_*SOLIDS*_ = solids of the curd as percentage of the solids of the milk processed; REC_*ENERGY*_ = energy of the curd as percentage of energy of the milk processed. ^1^True protein nitrogen (N) and milk N fractions are expressed as percentage of total milk N; β-CN (β-casein), κ-CN (κ-casein), αs_1_-CN (αs_1_-casein), αs_2_- CN (αs_2_-casein); caseins (CN): Σ(β-CN+κ-CN+ αs_1_-CN+αs_2_-CN); β-LG (β-lactoglobulin), α-LA (α-lactalbumin), whey proteins (WP): Σ(β-LG+ α-LA).

Variance components and heritability estimates from LAB and FIELD_*lact*1_ datasets for coagulation traits and milk acidity are reported in Table 3 and Figure 1. The coefficient of correlation for the calibration models developed on LAB measures (r_*C_LAB*_) and used for FIELD predictions, and the additive genetic correlation (r_*a*_) between LAB and FIELD traits are reported in Table 3 and Figure 2. As previously reported, in order to guarantee convergence of animal models, these analyses have been restricted to a dataset with only first lactation records of FIELD data. Generally, a decrease in both genetic and residual variances was observed in FIELD_*lact*1_ with respect to LAB data. A similar pattern was reported in other studies (Bonfatti et al., 2017). In particular, residual variances for LAB traits were 1.3–6 times greater than residual plus permanent environmental variances in FIELD_*lact*1_. In the case of LAB parameters obtained from mechanical lacto-dynamographs (traditional MCP and Curd firming model), this difference increases particularly for traits recorded at increasing time intervals from milk gelation. This is caused by the decreasing repeatability of the measures recorded by the instrument with the progress of the textural test. Therefore, heritability estimates were comparable between datasets for traits recorded earlier (e.g., RCT, k_*CF*_), whereas heritability estimates decreased for later measured LAB traits, but not for FTIR predicted FIELD_*lact*1_ traits (a_30_, a_45_, CF_*P*_, CF_*max*_). Optigraph, yielding an optical prediction and not a mechanical measurement, does not show the same decrease in repeatability and heritability, for traits describing the later part of CF pattern. Generally, heritability estimates of measured and predicted traits were comparable to other studies (Cecchinato et al., 2009; Bonfatti et al., 2017).

**TABLE 3:** Estimates of variance components and heritability of measured LAB traits, coefficient of correlation of the calibration model used for infrared prediction (r_*C_LAB*_) developed on LAB measures and used for FIELD predictions, estimates of variance components and heritability of Fourier transform infrared FIELD_*lact*1_ predictions, and additive genetic correlation (r_*a*_) between LAB and FIELD traits for the coagulation and acidity traits.

Traits^1^	LAB					r_*C_LAB*_	FIELD_*lact*1_^2^						r_*a*_	
			
	$\sigma_{\text{a}}^{2}$	$\sigma_{e}^{2}$	$\sigma_{p}^{2}$	h^2^	SE		$\sigma_{\text{a}}^{2}$	$\sigma_{pe}^{2}$	$\sigma_{e}^{2}$	$\sigma_{p}^{2}$	h^2^	SE	Est	SE
**Traditional MCP**														
RCT, min	7.155	14.967	22.122	0.323	0.098	0.858	5.911	2.091	8.901	16.904	0.350	0.003	0.892	0.081
k_20_, min	1.160	4.552	5.712	0.203	0.089	0.682	0.751	0.303	1.095	2.149	0.350	0.010	0.780	0.031
a_30_, mm	15.998	93.280	109.278	0.146	0.080	0.722	21.905	8.288	28.067	58.260	0.376	0.011	0.836	0.026
a_45_, mm	4.592	47.238	51.830	0.089	0.067	0.586	3.499	1.480	6.158	11.137	0.314	0.010	0.787	0.042
**Curd firming**														
RCT_*eq*_, min	6.648	15.588	22.236	0.299	0.098	0.853	6.057	2.301	10.469	18.827	0.322	0.010	0.863	0.018
CF_*p*_, mm	10.389	58.367	68.756	0.151	0.076	0.742	8.010	2.681	14.418	25.110	0.319	0.010	0.835	0.027
k_*CF*_, % × min^–1^	3.678	8.720	12.398	0.297	0.095	0.656	1.676	0.702	2.974	5.352	0.313	0.010	0.683	0.038
k_*SR*_, % × min^–1^	0.035	0.115	0.150	0.233	0.088	0.571	0.012	0.005	0.027	0.044	0.270	0.009	0.602	0.052
C_*max*_, mm	5.786	32.505	38.291	0.151	0.076	0.742	4.888	1.852	7.733	14.474	0.338	0.011	0.847	0.025
t_*max*_, min	21.794	54.064	75.858	0.287	0.097	0.774	18.862	7.647	29.049	55.558	0.340	0.011	0.783	0.028
**Optigraph**														
RCT, min	3.426	9.261	12.687	0.270	0.111	0.837	2.783	0.978	4.095	7.856	0.354	0.010	0.904	0.134
k_20_, min	1.629	4.441	6.070	0.268	0.119	0.809	1.426	0.466	1.567	3.460	0.412	0.011	0.919	0.012
a_30_, mm	26.076	76.993	103.069	0.253	0.115	0.760	20.611	8.158	28.236	57.005	0.362	0.003	0.802	0.016
a_45_, mm	47.194	64.650	111.844	0.422	0.131	0.743	19.957	7.373	28.803	56.133	0.356	0.010	0.799	0.024
**Acidity**														
pH	0.001	0.002	0.003	0.201	0.081	0.752	0.001	0.000	0.002	0.003	0.205	0.007	0.756	0.035

*^1^RCT = rennet coagulation time; k_20_ = curd firming rate as the time to a curd firmness of 20 mm; a_30 (45)_ = curd firmness at 30 (45) min from rennet addition; RCT_eq_ = rennet coagulation time estimated using the equation; CF_P_ = asymptotic potential curd firmness; k_CF_ = curd firming instant rate constant; k_SR_ = syneresis instant rate constant; CF_max_ = maximum curd firmness achieved within 45 min; t_max_ = time at achievement of CF_max_. ^2^FIELD_lact1_ = dataset limited to cows belonging to first lactation.*

*Cheese-making traits.* Variance components and heritability estimates of LAB and FIELD_*lact*1_ for cheese yield and recovery traits are reported in Table 4 and Figure 1. In addition, the r_*C_LAB*_ developed on LAB data and the r_*a*_ between LAB and FIELD traits are reported in Table 4 and Figure 2. For CY traits, genetic variances were larger in FIELD_*lact*1_ for CY_*CURD*_ (∼1.2-fold) and CY_*SOLIDS*_ (∼1.6-fold) and smaller for CY_*WATER*_ (∼1.2-fold). For all these traits, a ∼1.5-fold reduction was observed in residual plus permanent environmental variances. For CY_*CURD*_ and CY_*WATER*_, heritability estimates were comparable between the two datasets, while the heritability estimate of CY_*SOLIDS*_ was almost double in FIELD_*lact*1_ (0.358), compared to LAB (0.192). Variance components for REC traits had different behaviors: REC_*PROTEIN*_ and REC_*FAT*_ had smaller genetic variances for FIELD_*lact*1_ compared to LAB traits (∼1.6- and 1.2-fold, respectively), while REC_*SOLIDS*_ and REC_*ENERGY*_ had larger genetic variances in FIELD_*lact*1_, compared to LAB (∼1.3-fold). On the other hand, we observed a reduction in residual variance for all traits except REC_*PROTEIN*_ which had a slightly higher value in FIELD_*lact*1_, compared to the LAB trait. Accordingly, heritability estimates for REC traits were comparable or higher in FIELD_*lact*1_, with the exception of REC_*PROTEIN*_ which had a lower value in FIELD_*lact*1_ (0.247 vs. 0.563).

**TABLE 4:** Estimates (Est, with standard error reported as SE) of variance components and heritability of measured LAB traits, coefficient of correlation of the calibration model used for infrared prediction (r_*C_LAB*_) developed on LAB measures and used for predictions using FIELD spectra, estimates of variance components and heritability of Fourier transform infrared FIELD_*lact*1_ predictions, and additive genetic correlation (r_*a*_) between LAB and FIELD for the cheese yield, curd nutrient recovery traits, and protein fractions.

Traits^1^	LAB					r_*C_LAB*_	FIELD_*lact*1_^2^						r_*a*_	
			
	$\sigma_{\text{a}}^{2}$	$\sigma_{e}^{2}$	$\sigma_{p}^{2}$	h^2^	SE		$\sigma_{\text{a}}^{2}$	$\sigma_{pe}^{2}$	$\sigma_{e}^{2}$	$\sigma_{p}^{2}$	h^2^	SE	Est	SE
**Cheese yields, %**														
%CY_*CURD*_	0.408	1.687	2.094	0.195	0.079	0.899	0.495	0.218	1.075	1.788	0.277	0.010	0.814	0.028
%CY_*SOLIDS*_	0.105	0.443	0.548	0.192	0.080	0.905	0.170	0.066	0.240	0.477	0.358	0.011	0.754	0.034
%CY_*WATER*_	0.192	0.681	0.874	0.220	0.086	0.773	0.158	0.093	0.480	0.731	0.216	0.009	0.900	0.017
**Recoveries, %**														
REC_*PROTEIN*_	2.302	1.783	4.084	0.563	0.116	0.897	1.437	0.507	2.203	4.147	0.347	0.010	0.901	0.010
REC_*FAT*_	1.633	5.718	7.351	0.222	0.088	0.655	1.395	0.538	4.212	6.145	0.227	0.009	0.810	0.030
REC_*SOLIDS*_	1.707	7.071	8.778	0.194	0.081	0.861	2.283	0.792	3.124	6.200	0.368	0.011	0.868	0.019
REC_*ENERGY*_	1.539	6.933	8.472	0.182	0.081	0.814	2.027	0.703	3.963	6.693	0.303	0.004	0.902	0.010
**N fractions, % total N**														
Caseins	0.133	0.589	0.722	0.182	0.086	0.953	0.174	0.031	1.211	1.416	0.123	0.006	0.665	0.059
β-CN	3.199	1.492	4.690	0.678	0.104	0.656	0.519	0.180	1.450	2.148	0.242	0.009	0.782	0.020
κ-CN	0.765	0.752	1.516	0.501	0.106	0.687	0.300	0.170	1.145	1.615	0.186	0.008	0.760	0.029
αs1-CN	0.873	0.901	1.774	0.492	0.108	0.598	0.065	0.028	0.259	0.352	0.186	0.008	0.396	0.058
αs_2_-CN	0.240	0.466	0.706	0.337	0.104	0.623	0.056	0.033	0.187	0.276	0.203	0.009	0.535	0.058
Whey proteins	0.852	0.631	1.482	0.571	0.852	0.686	0.295	0.124	0.701	1.120	0.264	0.090	0.603	0.112
β-LG	0.635	0.537	1.712	0.538	0.103	0.722	0.289	0.117	0.976	1.382	0.209	0.008	0.656	0.034
α-LA	0.026	0.069	0.095	0.270	0.099	0.743	0.006	0.002	0.120	0.127	0.051	0.002	0.733	0.037

*^1^%CY_CURD_ = weight of fresh curd as percentage of weight of milk processed; %CY_SOLIDS_ = weight of curd solids as percentage of weight of milk processed; %CY_WATER_ = weight of water curd as percentage of weight of milk processed; REC_PROTEIN_ = protein of the curd as percentage of the protein of the milk processed; REC_FAT_ = fat of the curd as percentage of the fat of the milk processed; REC_SOLIDS_ = solids of the curd as percentage of the solids of the milk processed; REC_ENERGY_ = energy of the curd as percentage of energy of the milk processed; true protein nitrogen (N) and milk N fractions are expressed as percentage of total milk N; β-CN (β-casein), κ-CN (κ-casein), αs_1_-CN (αs_1_-casein), αs_2_-CN (αs_2_-casein); caseins: Σ(β-CN+κ-CN+ αs_1_-CN+αs_2_-CN); β-LG (β-lactoglobulin), α-LA (α-lactalbumin), whey proteins: Σ(β-LG+ α-LA). ^2^FIELD_lact1_ = dataset limited to cows belonging to first lactation.*

#### Milk Protein Fractions

Genetic parameters of milk protein fractions are also presented in Table 4 and Figure 1. In the case of milk proteins, heritability estimates of LAB traits were comparable to previous studies (Schopen et al., 2009). FTIR-predicted milk proteins showed a marked decrease in genetic variance, compared to LAB measures (especially α_*S*1_-CN, from 0.873 to 0.065), while residual variances were larger for total CN, κ-CN, total whey, β-LG, and α-LA, smaller for α_*S*1_-CN and α_*S*2_-CN, and comparable for β-CN. Heritability estimates were lower in FIELD_*lact*1_ for all protein traits, ranging from high-moderate (ranging from 0.182 to 0.678) to moderate-low (ranging from 0.051 to 0.264; Figure 1). These results were in accordance with previous studies, showing a decrease in the estimated genetic variance and heritabilities for predicted milk proteins compared to measured traits (Rutten et al., 2011; Bonfatti et al., 2017), and are essentially due to the low r_*C_LAB*_ values obtained for these traits.

### Reliability of Calibrations and Genetic Correlation Between Measured and Predicted Traits

**FIGURE 3:**
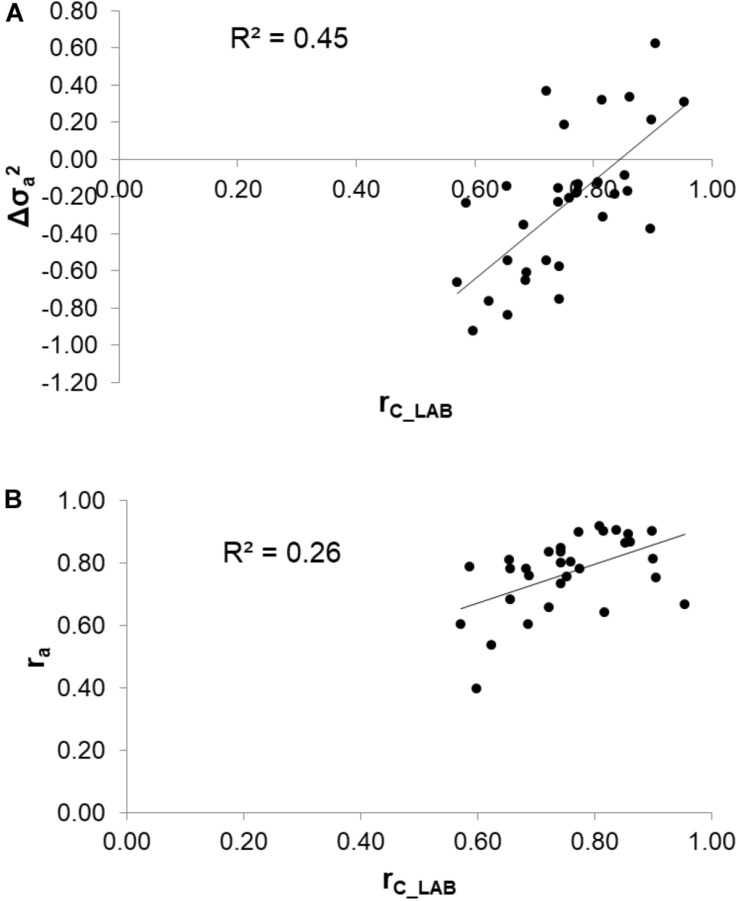
Relationships between the coefficient of correlation of calibration models (r_*C_LAB*_) used for infrared prediction and **(A)** the change in additive genetic variance ($\Delta \sigma_{a}^{2}$) and **(B)** the additive genetic correlation (r_*a*_) between LAB measures and FTIR predictions.

To assess the reliability of calibration equations in the animal breeding context, we estimated, besides the r_*C_LAB*_, the r_*a*_ between LAB and FIELD_*lact*1_ traits as an indicator of the potential of FTIR calibrations to provide novel phenotypes for indirect selective breeding. Results are displayed in Figure 2. The r_*C_LAB*_ ranged from 0.571 (k_*SR*_) to 0.953 (total CN). In general, estimates of r_*a*_ for milk coagulation and cheese-making traits were high (>0.75), except for k_*CF*_ and k_*SR*_ which displayed moderate-high estimates (>0.60). On the other hand, only two milk proteins showed r_*a*_ smaller than 0.6: α_*S*1_-CN (0.396) and α_*S*2_-CN (0.535). These two traits were those with the lowest r_*C_LAB*_ among all traits tested (Table 4: 0.598 and 0.623, respectively). This means that expected genetic gain using these two predicted milk proteins would be moderately lower, compared to that achievable for the other traits. These results were in accordance with Bonfatti et al. (2017) which found high r_*a*_ for milk protein content but lower r_*a*_ for percentage traits (0.58, on average). On the other hand, Rutten et al. (2011) reported greater r_*a*_ values compared to those in our study (0.62–0.97), which might be ascribed to differences in population (breed, size) and/or in the analytical technique used for milk protein investigation (capillary electrophoresis vs. HPLC). The reliability of calibration r_*C_LAB*_ was associated with the decrease in genetic variance between LAB and FIELD_*lact*1_ traits (*R*^2^ = 0.45). In particular, lower r_*C_LAB*_ corresponded to larger decrease in genetic variance (Figure 3A). A weaker relationship was observed between r_*C_LAB*_ and the decrease in residual variance (*R*^2^ = 0.09, data not reported in Figures). A positive relationship (*R*^2^ = 0.26) was observed between r_*C_LAB*_ and r_*a*_: a higher reliability of the calibration model corresponded to a higher additive genetic correlation between the LAB-measures and FIELD_*lact*1_ predictions (Figure 3B). Beyond this correlation, it is worth noting from Figure 2 that 22 out of 30 investigated traits presented genetic correlations greater than the reliability of calibration (r_*a*_ > r_*C_LAB*_). So, even calibrations with a relatively small predictive ability could be exploited in selective breeding, if their r_*a*_ between measured and predicted traits is sufficiently high.

### Single-Step GBLUP Prediction Accuracy by Cross-Validation

Table 5 and Supplementary Figure S1 report the results for the cross-validation performed in order to compare different phenotyping strategies. The cross-validation mimicked the progeny testing scheme, where the LAB phenotypes of testing sires were predicted based on LAB phenotypes from daughters of proven bulls and FIELD information from daughters of proven and progeny testing bulls. Results are presented as the average of the prediction accuracy for the fourfold of sires. For the scenarios that included FIELD information, results in Table 5 show the largest predictive ability obtained within the range of genetic correlation values tested, which is also reported in Table 5. The change in prediction accuracy for each trait over the values of genetic correlation is reported in Supplementary Figure S1.

**TABLE 5:** Best-performing model in terms of accuracy (acc) for each of the scenarios studied. The values in bold indicate the best performing scenario (in terms of accuracy) for each trait.

Trait^2^	LAB.t^1^	LAB.t + FIELD.t		LAB.t + FIELD.t + FIELD.v		FIELD.t		FIELD.t + FIELD.v	
	acc	r_*a*_	acc	r_*a*_	Acc	r_*a*_	acc	r_*a*_	acc
**Traditional MCP**									
RCT, min	0.118	0.9	0.285	**0.9**	**0.322**	0.9	0.285	0.9	0.321
k_20_, min	0.016	0.9	0.208	1.0	0.227	0.9	0.212	**0.4**	**0.233**
a_30_, mm	0.032	0.9	0.190	0.9	0.212	0.8	0.193	**0.9**	**0.215**
a_45_, mm	0.027	0.9	0.181	0.9	0.192	0.9	0.184	**0.9**	**0.196**
**Curd firming**									
RCT_*eq*_, min	0.102	0.9	0.286	**0.9**	**0.319**	0.9	0.286	0.9	0.312
CF_*p*_, mm	0.038	0.9	0.235	0.9	0.258	0.9	0.236	**0.9**	**0.262**
k_*CF*_, % × min^–1^	0.104	0.7	0.217	0.8	0.236	0.1	0.200	**1.0**	**0.249**
k_*SR*_, % × min^–1^	0.093	0.6	0.189	0.6	0.204	0.2	0.176	**0.2**	**0.216**
C_*max*_, mm	0.038	0.9	0.224	0.9	0.249	0.9	0.225	**0.9**	**0.258**
t_*max*_, min	0.112	0.9	0.233	**0.9**	**0.269**	0.9	0.223	0.9	0.265
Optigraph									
RCT, min	0.168	0.9	0.283	0.9	0.315	0.8	0.283	**0.8**	**0.316**
k_20_, min	0.053	1	0.250	**1.0**	**0.302**	0.9	0.248	1.0	0.296
a_30_, mm	0.037	0.9	0.242	0.9	0.262	0.9	0.257	**0.9**	**0.274**
a_45_, mm	0.060	0.9	0.331	**0.9**	**0.350**	0.9	0.325	0.9	0.346
Acidity									
pH	0.113	0.7	0.221	0.6	0.225	0.3	0.227	**0.3**	**0.236**
**Cheese yields, %**									
CY_*CURD*_	0.070	0.9	0.296	**0.9**	**0.317**	0.8	0.298	0.9	0.315
CY_*SOLIDS*_	0.088	0.9	0.348	**0.9**	**0.362**	0.9	0.343	0.9	0.331
CY_*WATER*_	0.076	0.9	0.203	0.9	0.242	0.9	0.198	**0.9**	**0.249**
Recoveries, %									
REC_*PROTEIN*_	0.147	1	0.340	**1**	**0.365**	1	0.329	1	0.362
REC_*FAT*_	0.078	0.9	0.192	**0.9**	**0.193**	0.9	0.192	0.9	0.182
REC_*SOLIDS*_	0.071	0.9	0.376	**0.9**	**0.377**	0.8	0.376	0.9	0.345
REC_*ENERGY*_	0.103	0.9	0.343	**0.9**	**0.358**	0.9	0.343	0.9	0.334
**N fractions, % total milk N**									
Caseins	0.036	0.9	0.198	**1**	**0.230**	0.9	0.200	1	0.204
β-CN	0.153	0.9	0.297	**0.9**	**0.308**	0.2	0.287	0.9	0.284
κ-CN	0.051	1	0.307	**1**	**0.330**	1	0.296	0.9	0.280
α_*S*1_-CN	**0.165**	0.1	0.164	0.1	0.165	0.8	0.055	0.8	0.061
α_*S*2_-CN	0.099	**0.6**	**0.170**	0.6	0.159	0.5	0.146	0.6	0.123
Whey proteins	0.208	0.7	0.243	0.5	0.238	0.9	0.206	**0.9**	**0.244**
β-LG	0.187	0.5	0.224	0.5	0.224	0.9	0.206	**0.9**	**0.254**
α-LA	0.054	0.9	0.140	0.9	0.137	0.5	0.133	**1**	**0.154**

*^1^The value of genetic correlation was implicitly considered equal to 0 in the LAB.t scenario. ^2^RCT = rennet coagulation time; k_20_ = curd firming rate as the time to a curd firmness of 20 mm; a_30 (45)_ = curd firmness at 30 (45) min from rennet addition; RCT_eq_ = rennet coagulation time estimated using the equation; CF_P_ = asymptotic potential curd firmness; k_CF_ = curd firming instant rate constant; k_SR_ = syneresis instant rate constant; CF_max_ = maximum curd firmness achieved within 45 min; t_max_ = time at achievement of CF_max_; %CY_CURD_ = weight of fresh curd as percentage of weight of milk processed; %CY_SOLIDS_ = weight of curd solids as percentage of weight of milk processed; %CY_WATER_ = weight of water curd as percentage of weight of milk processed; REC_PROTEIN_ = protein of the curd as percentage of the protein of the milk processed; REC_FAT_ = fat of the curd as percentage of the fat of the milk processed; REC_SOLIDS_ = solids of the curd as percentage of the solids of the milk processed; REC_ENERGY_ = energy of the curd as percentage of energy of the milk processed. ^1^True protein nitrogen (N) and milk N fractions are expressed as percentage of total milk N; β-CN (β-casein), κ-CN (κ-casein), αs_1_-CN (αs_1_-casein), αs_2_-CN (αs_2_-casein); caseins: Σ(β-CN+κ-CN+ αs_1_-CN+αs_2_-CN); β-LG (β-lactoglobulin), α-LA (α-lactalbumin), and whey proteins: Σ(β-LG+ α-LA).*

#### Prediction With LAB Information

In the LAB.t scenario, daughters of progeny testing bulls are not phenotyped and FIELD information is not included and the genetic evaluation is based on a single-trait model. The predictive ability for the different traits followed, to a large extent, the trait heritability (Figure 1). Among MCP traits, RCT showed the highest accuracy (0.118), with the other traits showing almost null values. For CF traits, only RCT_*eq*_, k_*CF*_, and t_*max*_ showed values of accuracy above 0.10. CF_*p*_ and C_*max*_ showed null values, which is in agreement with the low (∼0.1) heritability estimates. Optigraph traits showed a different trend, with RCT reporting the strongest prediction accuracy (0.168) but not the largest heritability. Prediction accuracies were below 0.1 for all CY traits, for which heritability estimates barely reached the value of 0.2. Prediction accuracy was largest for REC_*PROTEIN*_ among recovery traits, and its heritability estimate was the largest in the group (∼0.55). A similar scenario was found among casein fractions, where whey proteins, β-LG, and β-CN showed one of the strongest prediction accuracies (0.208, 0.187, and 0.153, respectively) and the largest heritability (above 0.50). Surprisingly, α_*S*1_-CN showed a strong predictive ability (0.165), yet not the largest heritability estimate (∼0.50). Prediction with only LAB information can be considered unreliable from a genetic evaluation standpoint, given the limited size of the dataset and the relative cost needed to acquire each single record. However, this scenario was included as a reference for the other, more reliable, models.

#### Prediction When LAB and FIELD Information Is Included

The LAB.t+FIELD.t scenario mimics a genetic evaluation where both LAB and FIELD information of proven bulls is included. The comparison with the LAB.t scenario proves the value of including FIELD.t information, which highlights the importance of the construction of FTIR calibration equations and the collection of data at the population level on daughters of proven bulls. Still, the genetic merit of bulls can be predicted at birth under this scenario. The genetic evaluation leverages a bivariate model. Overall, prediction accuracy showed a strong increase, compared to the previous scenario. The largest increases in prediction accuracy were observed for curd firming traits, with MCP k_20_, a_30_, and a_45_ having prediction accuracies of 0.208, 0.190, and 0.181, respectively (compared to null accuracies in LAB.t); Optigraph a_30_ prediction accuracy increased from 0.037 to 0.242; and CF_*p*_ increased from 0.038 to 0.235. Among protein composition traits, κ-CN increased from 0.051 to 0.330, while α_*S*1_-CN did not increase (being the only trait not showing any increase). For all the abovementioned traits that showed an increase, the optimal value of genetic correlation appeared to be 0.9, which corroborates the relevance of FIELD information for accurately predicting LAB breeding values and phenotypes. The values of correlation for the calibration model used for infrared prediction (r_*C_LAB*_) and additive genetic correlations (r_*a*_) estimated from the data were large for these traits but not the largest found.

The LAB.t+FIELD.t+FIELD.v scenario mimicked a genetic evaluation based on a progeny testing scheme, which implies the collection of FTIR spectra information on the daughters of progeny testing bulls (FIELD.v). Here, genetic merit of bulls cannot be estimated at birth but collection of FIELD phenotypes is needed as in traditional progeny testing. As compared to a previously discussed scenario, the advantage of the FIELD.v data seemed marginal, showing a maximum of 1.2-fold increase and often a decrease in prediction accuracy. Traits that benefited the most were OPT k_20_ (from 0.250 to 0.302), CY_*WATER*_ (from 0.203 to 0.242), and casein proportion (from 0.198 to 0.230). Traits that did not show an increase were α_*S*2_-CN, whey proteins, α-LA, and β-LG.

#### Prediction When LAB Information Is Not Included

Scenarios FIELD.t and FIELD.t+FIELD.v mimic a breeding scheme where LAB information is not included in the genetic evaluations. Still, LAB information is used to obtain FTIR phenotype prediction equations. The underlying model is a bivariate model that produces LAB breeding values, although LAB records are not included. Comparing the LAB.t+FIELD.t scenario to the FIELD.t scenario allows the evaluation of the contribution of LAB information when FIELD information is recorded on the daughters of proven bulls. Here, differences were negligible, except for α_*S*1_-CN that showed a dramatic decrease from 0.165 to 0.055 when LAB information was removed. This is due to the low quality of the calibration model used for FTIR predictions (r_*C_LAB*_ = 0.60 and r_*a*_ = 0.40). The comparison of the LAB.t+FIELD.t+FIELD.v scenario to the FIELD.t+FIELD.v allows us to further prove the value of LAB phenotypes when FIELD information is also recorded in daughters of progeny testing bulls. Again, changes were negligible for most traits, confirming the low relevance of LAB information when solid FTIR calibration equations are used. The relevance of FIELD.v information is assessed in the comparison of FIELD.t and FIELD.t+FIELD.v, when LAB information is absent. While the increase was negligible in the presence of LAB information (maximum of 1.2-fold increase in accuracy), in this case the fold increase reached 1.25 with several traits being about 1.1. This supports the hypothesis that progeny testing could be beneficial for most traits, but probably the gain in accuracy would not match the cost of delaying the candidate bull’s evaluation. It should be considered that generation interval can be dramatically reduced when FIELD.v information is omitted, compromising prediction accuracy, but probably not sufficiently to justify progeny testing cost.

#### Best Performing Scenario in Terms of Prediction Accuracy

With the exception of α_*S*1_-CN, which showed a poor FTIR calibration equation, all traits showed an advantage in the inclusion of FIELD information, supporting the need for well-constructed calibration equations that allow us to obtain predicted phenotypes at the population level. Furthermore, an increase in prediction accuracy was found with the inclusion of FIELD.v information rather than just FIELD.t, either with or without the presence of LAB.t phenotypes. Scenarios that included all FIELD information outperformed scenarios that just included FIELD.t, with the exception of α_*S*2_-CN. This would lead to further speculation on the need for conducting progeny testing for new bulls, but the gain in accuracy of prediction is probably not translated into gain in genetic progress due to increased generation interval. It should also be noted that for some traits, i.e., k_20_, k_*CF*_, k_*SR*_, βCN, pH, α_*S*1_-CN, and α-LA (not necessarily those with low FTIR prediction accuracy), the trend of prediction accuracy over the values of assumed genetic correlation was sometimes null if not negative.

Despite the large number of traits involved, this study does not allow us to declare a breakeven value for any genetic parameter that would serve to predict the value of FIELD vs. LAB information. The only trait that did not benefit from FIELD information happened to be α_*S*1_-CN, for which the quality of the FTIR calibration equation was particularly low, followed by the low FIELD heritability and low genetic correlation with LAB measures. All other traits benefited from the inclusion of FIELD information and the accuracy gained with FIELD information greatly depended on the genetic correlation between LAB and FIELD traits. Nonetheless, it was not possible to declare a breakeven value of genetic correlation which deemed the use of FIELD information advantageous. Breakeven values of genetic correlations for indicator traits were found to be 0.5 by Calus and Veerkamp (2011), who studied genomic selection performance under multi-trait scenarios, and 0.7–0.8 by Mulder and Bijma (2007) who studied the impact of genotype by environment interactions in breeding programs. Given that this study based on field data, there could be more factors affecting the value of indicator traits in genomic prediction. Further research is needed in order to explore all potential contributions.

## Conclusion

The present study reported two approaches for assessing the contribution of FTIR calibration equations at the population level for dairy cattle breeding. With the first approach, results indicate that FIELD predictions can be used in breeding programs for the genetic improvement of difficult-to-measure traits and that indirect selection for FIELD predictions will provide satisfactory responses. With the second approach, for the first time we highlighted the utility of FTIR predictions for breeding purposes using real data to simulate different genetic evaluation scenarios, where FTIR-derived phenotypic information is dosed into (single-step) GBLUP to predict wet-lab measured performance of daughters of progeny testing bulls. Collection of FIELD measures for progeny testing bulls appears to be advantageous for increasing the predictive ability for most of the traits studied, but the increase in generation interval due to progeny testing does not justify the increase in prediction accuracy. LAB information from proven bulls’ daughters could be included in the genetic evaluations without a detrimental effect. As there is no evidence of a clear advantage to including FIELD information for progeny testing bulls, progeny testing schemes could be replaced by the construction of robust calibration equations together with more vast collection of FIELD measures on daughters of proven bulls.

In general, the increase in predictive ability observed with the inclusion of FIELD information is very favorable and reaches moderate values for 12 traits. While further research is needed in the modeling of FTIR-predicted data, results are promising. Once calibration equations are developed, the cost of collecting FIELD is virtually null, provided that routine spectra acquisition within milk recording schemes is performed and available for breeders. Yet, the cost of developing robust calibration equations should be factored into the total cost of implementing (genomic) selection that includes FIELD data. Thus, an economic analysis should be performed before progressing its use in breeding programs for difficult-to-measure traits.

## Data Availability Statement

The raw data supporting the conclusions of this article will be made available by the authors, without undue reservation.

## Author Contributions

AC conceived the study, contributed to set up the objectives of this study, drafted the first version of the manuscript, and supervised the project. HT-A performed the statistical analysis together with FT. SP contributed to the critical interpretation of the results and to manuscript drafting. GB and CM contributed to the critical revision of the manuscript. FT conceived the study with AC, contributed to the critical interpretation of the results, and to manuscript drafting. All authors have read and approved the final manuscript.

## Conflict of Interest

The authors declare that the research was conducted in the absence of any commercial or financial relationships that could be construed as a potential conflict of interest.
