# Supplementary material for: Integration of Wet-Lab Measures, Milk Infrared Spectra, and Genomics to Improve Difficult-to-Measure Traits in Dairy Cattle Populations
Source: Front Genet. 2020 Sep 29;11:563393. doi: 10.3389/fgene.2020.563393 (PMC7550782; doi:10.3389/fgene.2020.563393)

**Table S1**. Number of sires, records and cows allocated in each validation fold. Each fold was alternatively removed from the data for model comparison by cross-validation.

|  | **Fold 1** | **Fold 2** | **Fold 3** | **Fold 4** | **Other^1^** |
| --- | --- | --- | --- | --- | --- |
| Number of sires^2^ | 44 | 45 | 44 | 48 | 1,654 |
| N – LAB records | 226 | 259 | 189 | 174 | 260 |
| N – FIELD records | 101,249 | 94,534 | 68,529 | 62,890 | 401,772 |
| N – FIELD cows | 6,335 | 6,186 | 4,493 | 4,439 | 29,594 |

^1^Sires, records and cows that were never masked but always entered the training set.

^2^Number of sires chosen for validation. The sires had to be genotypes and show progeny in both LAB and FIELD datasets.

**Table S2.** Variance components values estimated with univariate models and then used in the cross-validation.

| Trait^1^ | FIELD | | | | LAB | | |
| --- | --- | --- | --- | --- | --- | --- | --- |
|  | σ^2^_a_ | σ^2^_pe_ | σ^2^_e_ | σ^2^_a_ | | σ^2^_e_ |  |
| Traditional MCP |  |  |  |  | |  |  |
| RCT, min | 9.60 | 1.11 | 7.52 | 7.00 | | 15.62 |  |
| k_20_, min | 1.28 | 0.10 | 0.96 | 1.41 | | 4.28 |  |
| a_30_, mm | 34.87 | 3.53 | 25.81 | 18.46 | | 92.99 |  |
| a_45_, mm | 6.33 | 0.65 | 5.10 | 7.74 | | 44.11 |  |
| Curd firming |  |  |  |  | |  |  |
| RCT_eq_, min | 10.32 | 1.16 | 8.87 | 6.63 | | 16.05 |  |
| CF_p_, mm | 13.39 | 1.41 | 10.83 | 14.07 | | 55.84 |  |
| k_CF_, %×min^-1^ | 2.23 | 0.38 | 2.42 | 4.33 | | 8.20 |  |
| k_SR_, %×min^-1^ | 0.01 | 0.00 | 0.02 | 0.04 | | 0.11 |  |
| C_max_, mm | 8.77 | 0.85 | 6.67 | 7.81 | | 31.03 |  |
| t_max_, min | 30.62 | 3.57 | 26.17 | 21.25 | | 56.09 |  |
| Optigraph |  |  |  |  | |  |  |
| RCT, min | 4.18 | 0.60 | 3.55 | 3.94 | | 8.96 |  |
| k_20_, min | 2.07 | 0.23 | 1.40 | 2.03 | | 4.09 |  |
| a_30_, mm | 29.31 | 3.99 | 24.72 | 35.45 | | 69.22 |  |
| a_45_, mm | 29.63 | 2.99 | 25.94 | 51.49 | | 60.68 |  |
| Acidity |  |  |  |  | |  |  |
| pH | 0.00075 | 0.00015 | 0.00105 | 0.00085 | | 0.00243 |  |
| Cheese yields, % |  |  |  |  | |  |  |
| CY_CURD_ | 0.68 | 0.09 | 0.77 | 0.54 | | 1.57 |  |
| CY_SOLIDS_ | 0.27 | 0.03 | 0.28 | 0.12 | | 0.44 |  |
| CY_WATER_ | 0.20 | 0.04 | 0.23 | 0.23 | | 0.63 |  |
| Recoveries, % |  |  |  |  | |  |  |
| REC_PROTEIN_ | 1.71 | 0.33 | 1.59 | 2.06 | | 2.05 |  |
| REC_FAT_ | 1.73 | 0.31 | 2.76 | 2.41 | | 5.09 |  |
| REC_SOLIDS_ | 3.58 | 0.26 | 3.36 | 2.20 | | 6.64 |  |
| REC_ENERGY_ | 2.97 | 0.24 | 4.16 | 1.51 | | 6.97 |  |
| N fractions, % total milk N |  |  |  |  | |  |  |
| Caseins | 0.17 | 0.07 | 0.68 | 0.18 | | 0.59 |  |
| β- CN | 0.57 | 0.20 | 0.90 | 3.45 | | 1.47 |  |
| κ-CN | 0.28 | 0.10 | 0.65 | 0.74 | | 0.78 |  |
| α_S1_-CN | 0.09 | 0.03 | 0.23 | 0.98 | | 0.85 |  |
| α_S2_-CN | 0.08 | 0.02 | 0.14 | 0.22 | | 0.49 |  |
| Whey proteins | 0.33 | 0.05 | 0.33 | 0.81 | | 0.66 |  |
| β-LG | 0.30 | 0.06 | 0.36 | 0.63 | | 0.53 |  |
| α-LA | 0.01 | 0.00 | 0.04 | 0.03 | | 0.07 |  |

^1^RCT = rennet coagulation time; k_20_ = curd firming rate as the time to a curd firmness of 20 mm; a_30 (45)_ = curd firmness at 30 (45) min from rennet addition; RCT_eq_ = rennet coagulation time estimated using the equation; CF_P_ = asymptotic potential curd firmness; k_CF_ = curd firming instant rate constant; k_SR_ = syneresis instant rate constant; CF_max_ = maximum curd firmness achieved within 45 min; t_max_ = time at achievement of CF_max_; %CY_CURD_ = weight of fresh curd as percentage of weight of milk processed; %CY_SOLIDS_ = weight of curd solids as percentage of weight of milk processed; %CY_WATER_ = weight of water curd as percentage of weight of milk processed; REC_PROTEIN_ = protein of the curd as percentage of the protein of the milk processed; REC_FAT_ = fat of the curd as percentage of the fat of the milk processed; REC_SOLIDS_ = solids of the curd as percentage of the solids of the milk processed; REC_ENERGY_ = energy of the curd as percentage of energy of the milk processed; ^1^True protein nitrogen (N) and milk N fractions are expressed as percentage of total milk N; β-CN (β-casein), κ-CN (κ-casein), αs_1_-CN (αs_1_-casein), αs_2_- CN (αs_2_-casein); Caseins: ∑( β-CN+κ-CN+ αs_1_-CN+αs_2_-CN); β-LG (β-lactoglobulin), α-LA (α-lactalbumin), Whey proteins: ∑( β-LG+ α-LA).

**Supplementary material**

**Figure S1.** Predictive ability of different scenarios assessed using cross validation on the analyzed traits. a) Traditional milk coagulation properties (RCT = rennet coagulation time; k_20_ = curd firming (CF) rate as the time to a curd firmness of 20 mm; a_30_ (_45_) = curd firmness at 30 (_45_) min from rennet addition); b) Curd firming (RCT_eq_ = rennet coagulation time estimated using the equation; CF_P_ = asymptotic potential curd firmness; k_CF_ = curd firming instant rate constant; k_SR_ = syneresis instant rate constant; CF_max_ = maximum curd firmness achieved within 45 min; t_max_ = time at achievement of CF_max_); c) Optigraph d) Milk acidity; e) Cheese yields (CY= cheese yield; %CY_CURD_ = weight of fresh curd as percentage of weight of milk processed; %CY_SOLIDS_ = weight of curd solids as percentage of weight of milk processed; %CY_WATER_ = weight of water curd as percentage of weight of milk processed); f) Recoveries (REC= recoveries; REC_PROTEIN_ = protein of the curd as percentage of the protein of the milk processed; REC_FAT_ = fat of the curd as percentage of the fat of the milk processed; REC_SOLIDS_ = solids of the curd as percentage of the solids of the milk processed; REC_ENERGY_ = energy of the curd as percentage of energy of the milk processed); g) Milk N fractions (β-CN (β-casein), κ-CN (κ-casein), αs_1_-CN (αs_1_-casein), αs_2_- CN (αs_2_-casein); Caseins (CN): ∑( β-CN+κ-CN+ αs_1_-CN+αs_2_-CN); β-LG (β-lactoglobulin), α-LA (α-lactalbumin), Whey proteins (WP): ∑( β-LG+ α-LA). The y-axis reports the accuracy of prediction, the x-axis reports the assumed genetic correlation between LAB and FIELD measures. The black square reports the prediction accuracy of the LAB.t model. The grey circle reports the ‘FIELD.t’ model, the grey triangle reports results from the model ‘LAB.t + FIELD.t’, the black circles report the model ‘FIELD.t + FIELD.v’ and the black triangle reports the model ‘LAB.t + FIELD.t + FIELD.v’.


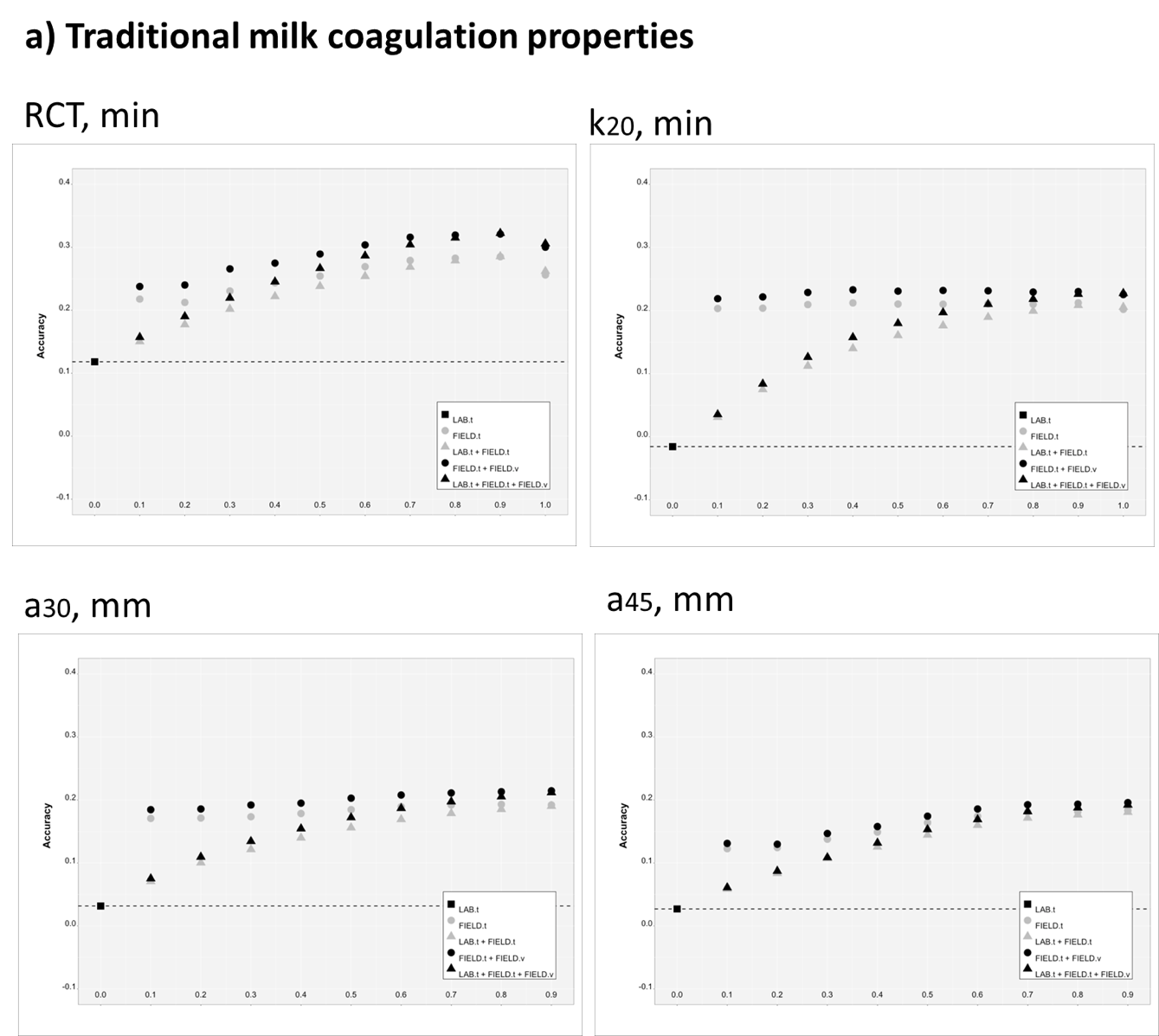


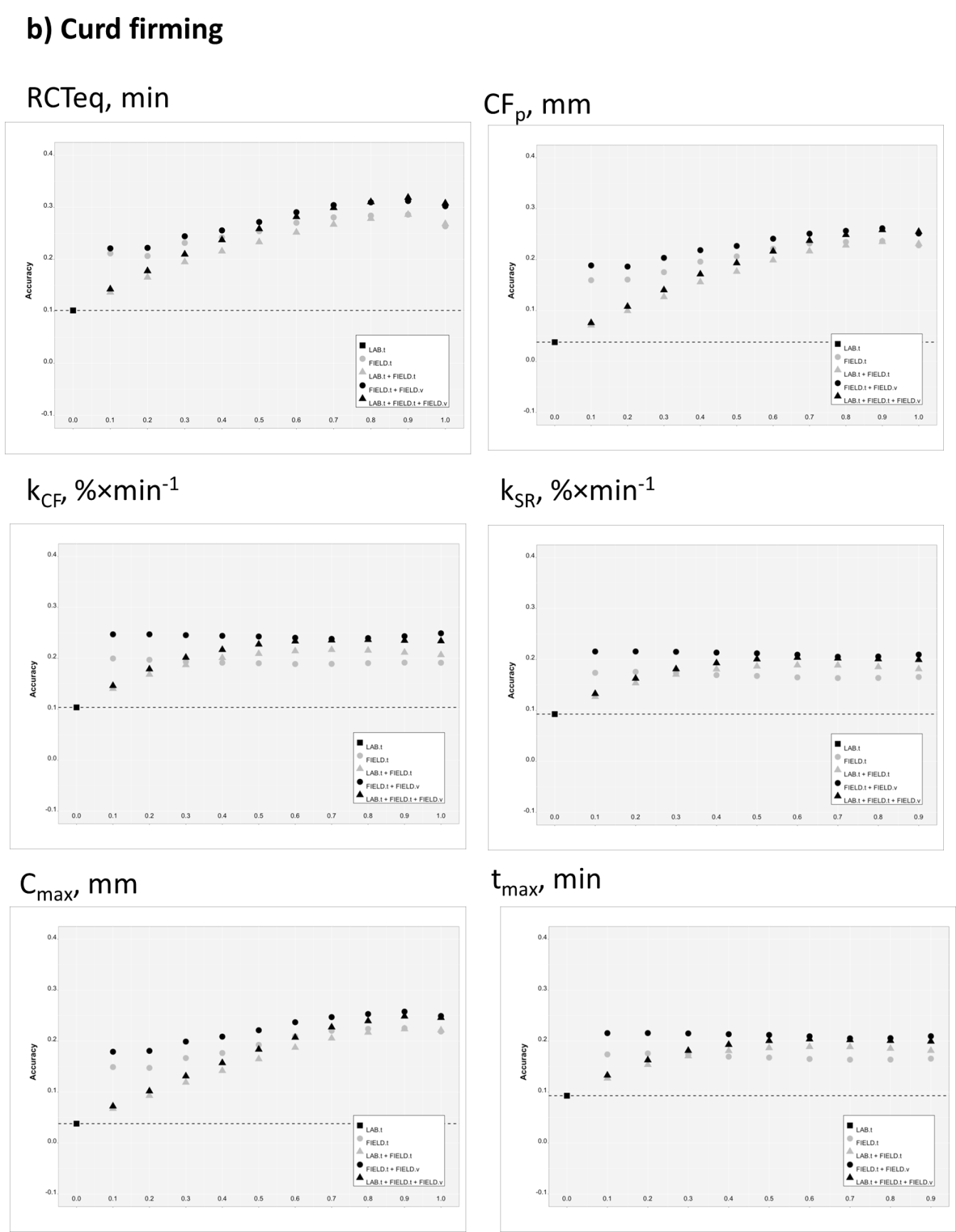


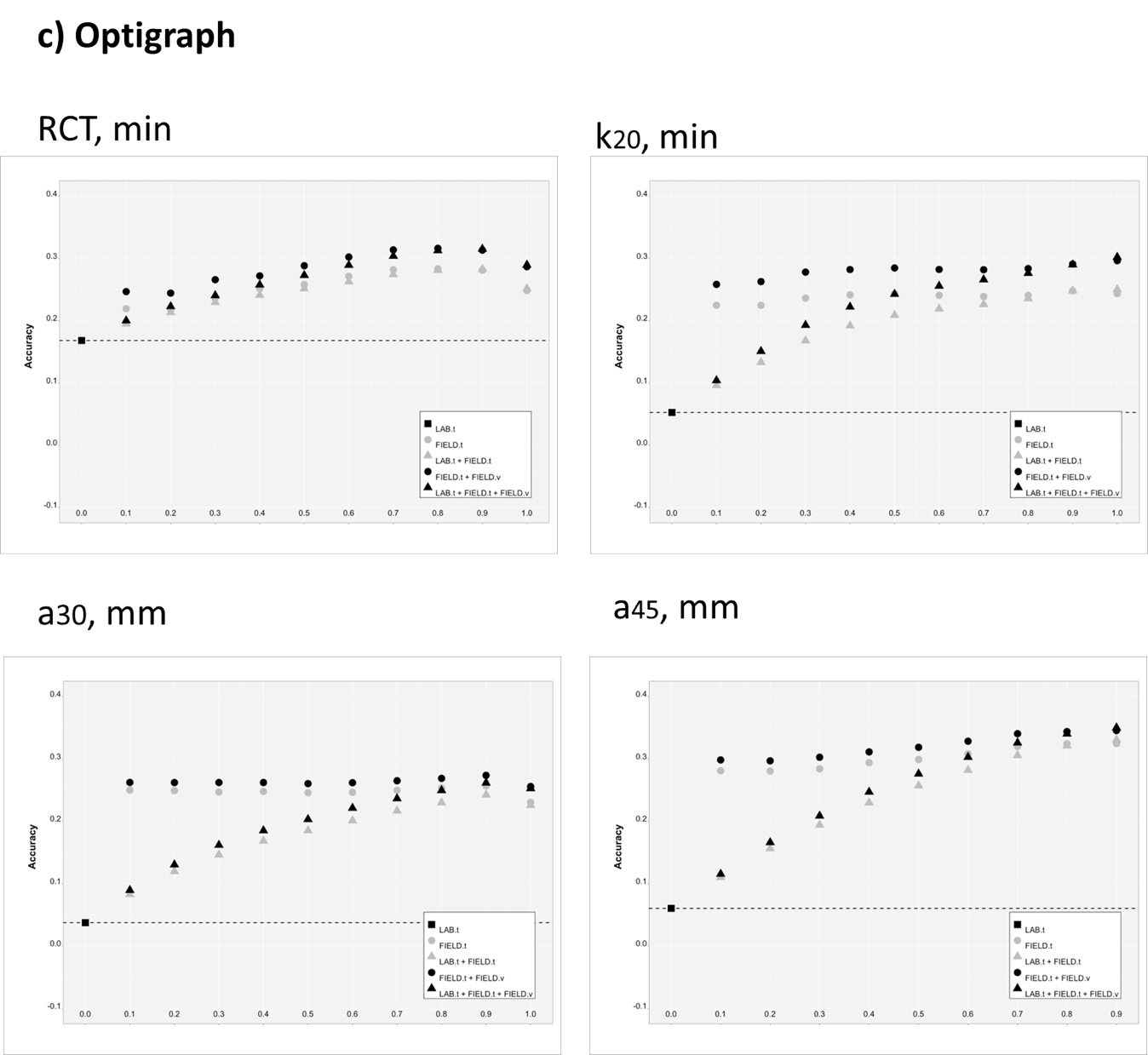


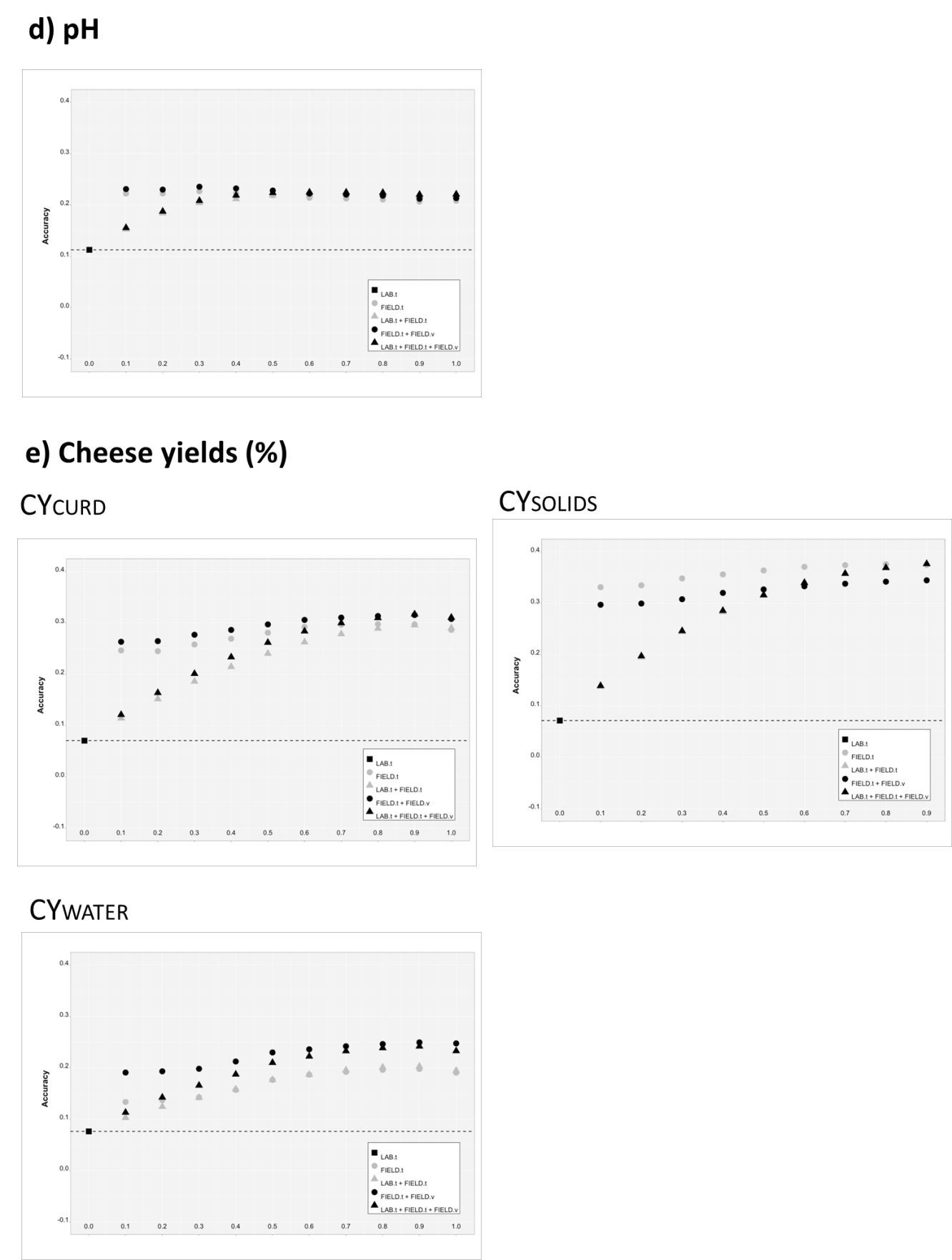


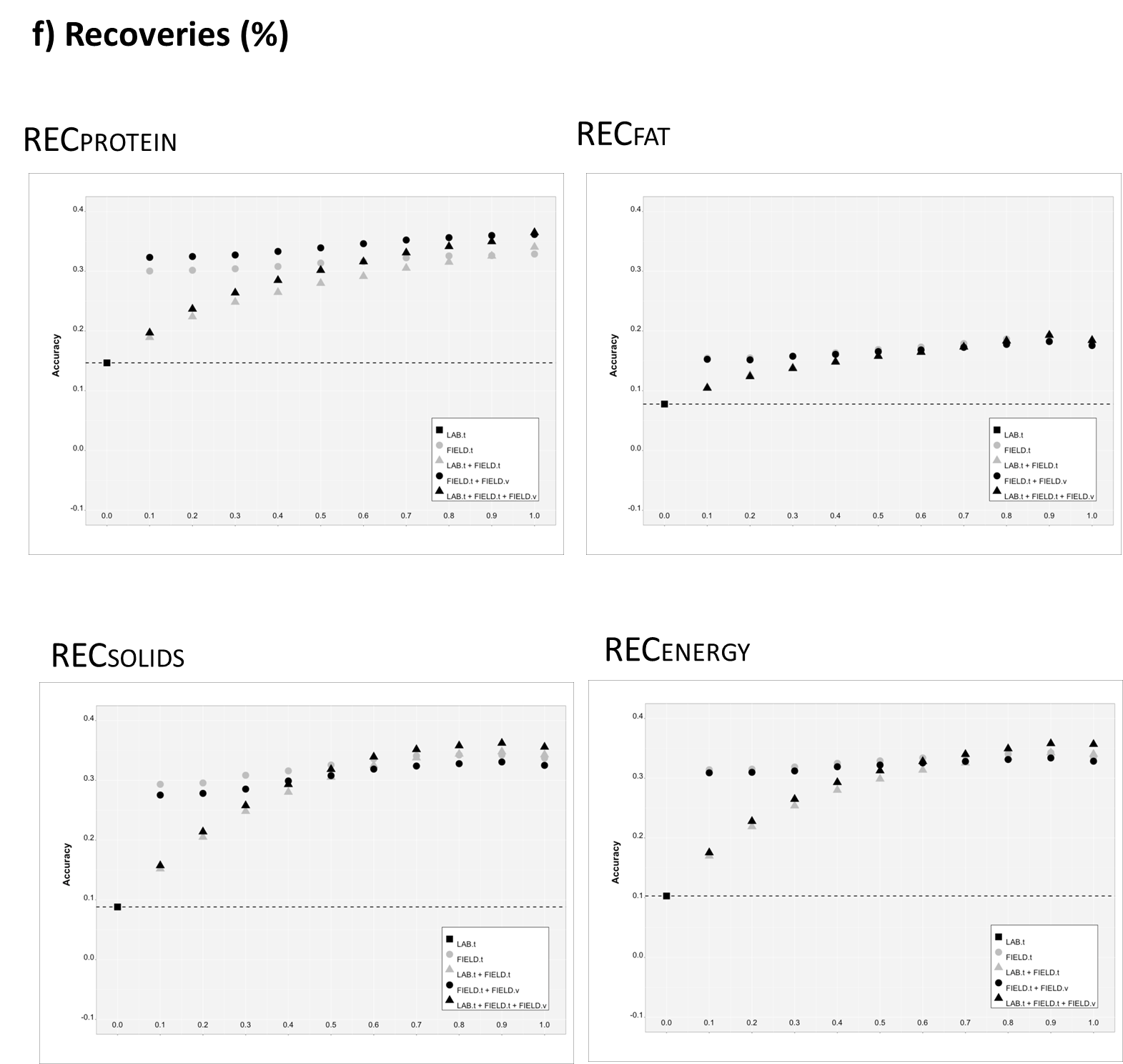


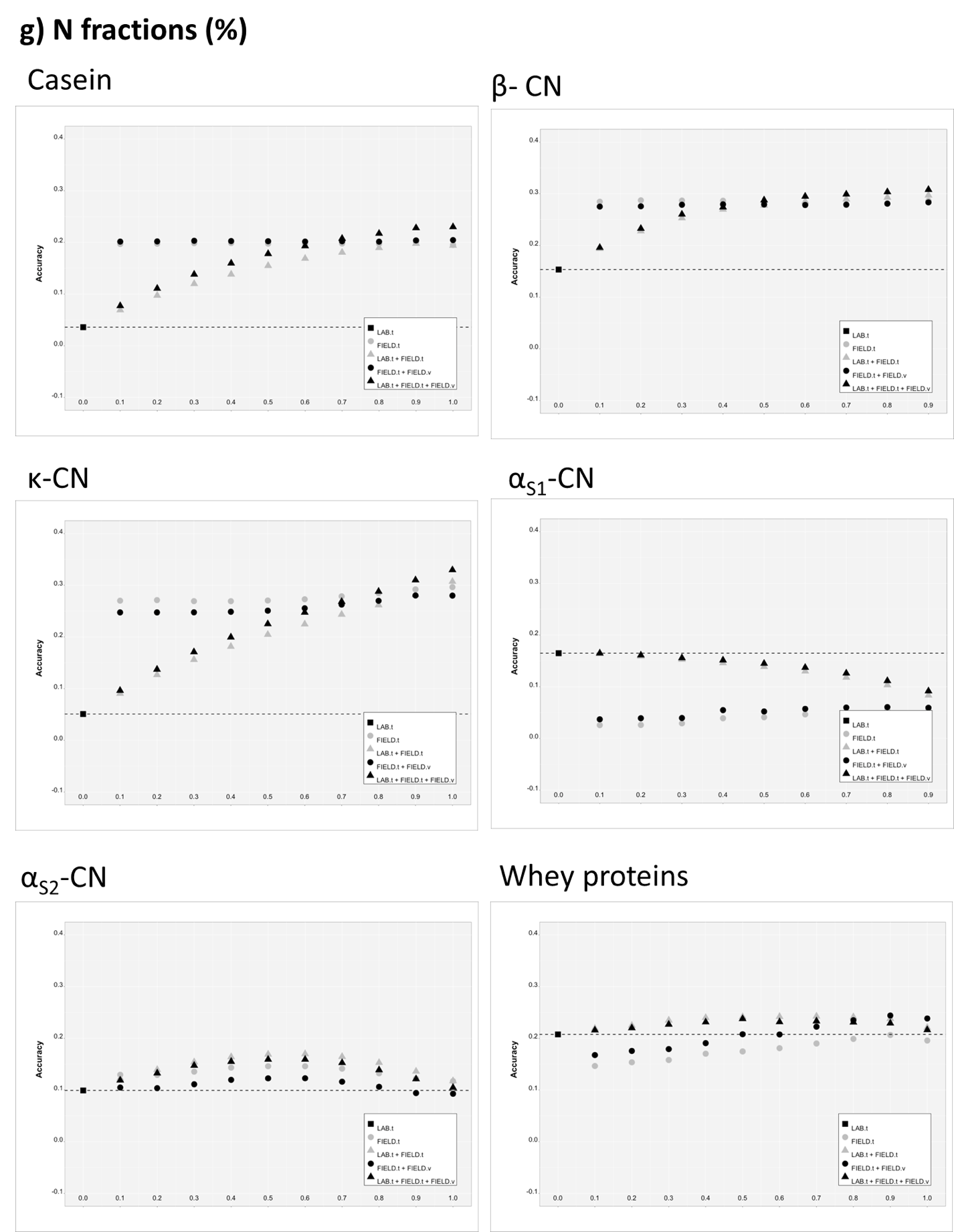


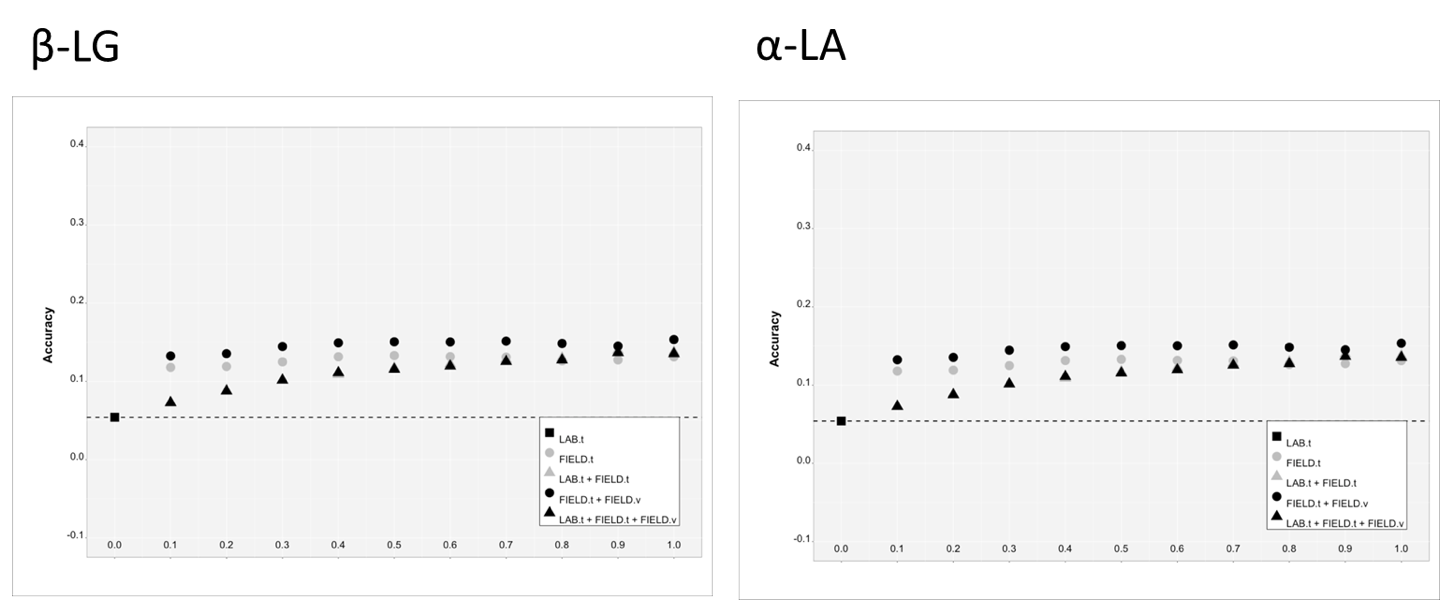

Supplement: Supplementary file 1 [file Data_Sheet_1.docx]
